# Structural basis of co-translational N-myristoylation in humans

**DOI:** 10.1038/s41467-025-67962-4

**Published:** 2026-01-23

**Authors:** Timo Denk, Paul Monassa, Joanna Musial, Otto Berninghausen, Birgitta Beatrix, Carmela Giglione, Thierry Meinnel, Roland Beckmann

**Affiliations:** 1 Gene Center and Department of Biochemistry, Feodor-Lynen-Str. 25, Munich, LMU Munich Germany; 2 Université Paris-Saclay, CEA, CNRS, Institute for Integrative Biology of the Cell (I2BC), Gif-sur-Yvette, France

**Keywords:** Cryoelectron microscopy, Ribosome

## Abstract

Modifications of proteins occurring during translation are critical for protein localization, stability and function. N-myristoylation is an essential N-terminal lipid modification catalyzed co-translationally by N-myristoyltransferases (NMTs) which have been identified as promising drug targets. However, its molecular basis in the context of the translating ribosome is not known. Here, we reveal the structural basis for co-translational N-myristoylation by NMT1 on the human ribosome by cryo-electron microscopy (cryo-EM). We show that NMT1 binds near the peptide tunnel exit and interacts with the nascent polypeptide-associated complex (NAC). Unlike other multi-enzyme complexes that act simultaneously, we find that methionine excision by methionine aminopeptidases and N-myristoylation occur sequentially via consecutive binding to the ribosome. Furthermore, our data suggest that NMT1 remains associated with elongating nascent chains, indicating a co-translational chaperone-like function in partnership with NAC. These insights provide a molecular foundation for the understanding of the co-translational N-myristoylation mechanism in humans.

## Abstract (web summary)

N-Myristoylation by NMT1 is an essential modification in humans safeguarding transient membrane anchorage of proteins. Here, the authors use cryo-EM to visualize the foundation for the co-translational modification activity of NMT1 on the ribosome.

## Introduction

Co-translational protein modifications are precisely orchestrated at the ribosome, where an array of enzymes ensures the functional and structural maturation of nascent polypeptide chains, safeguarding proteome integrity, targeting, and cellular homeostasis (reviewed in refs. ^1,2^). Among these modifications, N-terminal myristoylation (N-myristoylation) is an essential and irreversible lipidation reaction^3^ that profoundly influences membrane anchoring, governs subcellular trafficking, and interaction dynamics, thereby regulating various biological processes, including signaling pathways, immune responses, apoptosis, and pathogen-host interactions^3,4^. Substrates include proteins that play a major role in vesicular membrane trafficking like ADP-ribosylation factors (ARFs) or signal transduction like Src family tyrosine kinases and Gα subunits, amongst others^3,4^. As recently uncovered, N-myristoylation also contributes to protein stability as it masks N-degron motifs that conversely function as quality control for myristoylation fidelity^5^. Proteomics and biochemical studies have further expanded this catalog of predicted N-myristoylated proteins, revealing that approximately 2% of eukaryotic proteomes (~ 400 proteins in humans) undergo this lipid modification^3,6,7^.

N-myristoylation, the attachment of a 14-carbon saturated fatty acid myristoyl moiety, occurs predominantly co-translationally and at an N-terminal glycine residue that is typically exposed after removal of initiator methionine by methionine aminopeptidases (MetAPs)^8–10^. This reaction is catalyzed by N-myristoyltransferases (NMTs), conserved enzymes belonging to the GCN5-related N-acetyltransferase (GNAT) superfamily^3^. In metazoans, including humans, NMT activity is mediated by two highly similar isoforms, NMT1 and NMT2^11^, whereas only one NMT exists in unicellular eukaryotes like yeast^3^. NMT1 and NMT2 share high structural similarity yet possess nuanced functional divergences with NMT1 being the most abundant NMT by far (Supplementary Table 1)^3^. Conversely, deregulation of N-terminal myristoylation and especially NMT1 has substantial pathological consequences, implicated in cancers, viral infections, inflammation, and neurodegenerative disorders^12^. Since many pro-oncogenic pathways depend on myristoylation but are often not directly targetable, NMT1 inhibition has come into the focus of drug development^13,14^. Therefore, the mechanistic details of myristoyl transfer by human NMTs have been studied extensively over the years^14–16^. Remarkably, NMT1 - like MetAPs (METAP1 and METAP2 in human), and other competing catalysts such as the N-acetyltransferase NatA - is a highly abundant protein that accumulates in near-stoichiometric amounts with ribosomes, at concentrations comparable to other ribosome-associated factors such as the nascent polypeptide-associated complex (NAC; Supplementary Table 1). However, the precise mechanism by which NMT1 integrates into the dynamic environment of the translating ribosome, and how its co-translational activity is coordinated with that of both human MetAPs and other ribosome-associated enzymes remain unclear.

In this work, we unravel the structural foundation underlying human NMT1 activity at the ribosome using high-resolution cryo-electron microscopy of native and reconstituted ribosome-nascent chain complexes (RNCs). We show that MetAP and NMT1 nascent polypeptide engagement is sequential and mutually exclusive at the ribosome. Finally, we provide structural insights into a potential chaperone-like role of NMT1 together with NAC that broadens the role of NMT1 besides its essential myristoylation activity.

## Results

### Simultaneous ribosome association of NMT1 and NAC in vivo

**Fig. 1:**
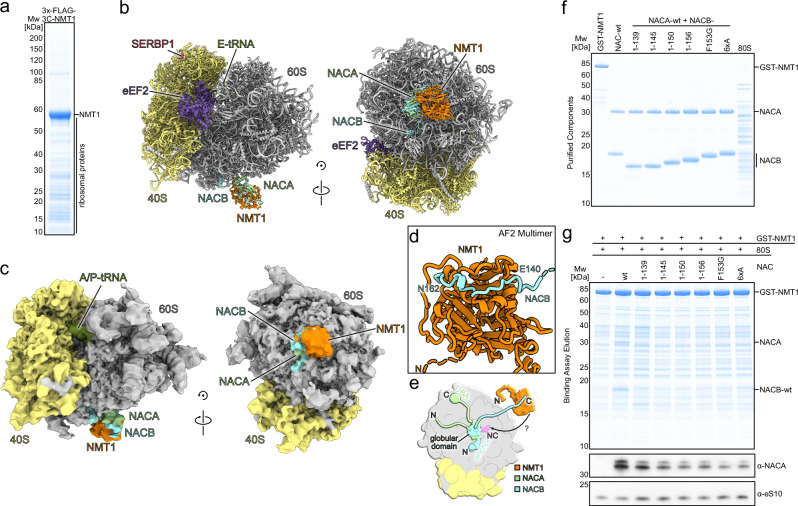
NMT1-ribosome association ex vivo. **a** SDS-PAGE gel stained with Coomassie of the elution fraction of tagged NMT1 purified under native conditions from Expi293F cells. **b** Molecular model for the combined translational states with NMT1-NAC bound. **c** Colored, local resolution filtered cryo-EM density of active hybrid (A/P P/E tRNA) 80S subclass from the native NMT1 purification. **d** Close-up view of the AF2 multimer prediction for the interaction of the NACB C-terminus with NMT1 numbered according to NACB isoform 2 used in (**f** and **g**). **e** Cartoon illustrating the overall domain organization of the NAC complex on the ribosome (40S yellow, 60S gray). The potential cooperativity with NMT1 in ribosome association via the NACB C-terminus in question is illustrated. **f** SDS-PAGE gel of purified components used for in vitro binding assay stained with Coomassie. **g** SDS-PAGE gel of elution fractions (50%) of in vitro NMT1-ribosome binding assay with different NAC complex mutants stained with Coomassie (top) and Western blots of elution fractions (5%) probed for NACA and ribosomal protein eS10 (bottom). Source data are provided as a Source Data file.

To analyze the NMT1 interaction with the translating ribosome in vivo, we first performed a polysome gradient analysis from cells overexpressing N-terminally 3xFLAG-tagged NMT1 with a 3C protease cleavage site. Consistently, tagged NMT1 co-migrated into the deep polysome fractions indicating ribosome association in vivo (Supplementary Fig. 1a, left), and overexpression had no substantial impact on the modified proteome of cellular lysates as assessed by employing click-chemistry to label NMT substrates (Supplementary Fig. 1a, right). We then purified tagged NMT1 expressed in Expi293F suspension cells under native conditions and indeed co-precipitated ribosomes (Fig. 1a). These isolated native ribosome complexes were stabilized by crosslinking with glutaraldehyde and subjected to cryogenic-electron microscopy single particle analysis (cryo-EM SPA). After 3D classification (Supplementary Fig. 2a–c) one fraction of particles represented actively translating 80S, whereas the larger fraction turned out to be hibernating, non-translating ribosome species, which is most likely due to overexpression of the tagged NMT1. Yet, regardless of the translational state, docking of molecular models revealed that all ribosomal particles showed distinct density on the 60S subunit for NMT1 and, unexpectedly, the NAC complex, a highly conserved ribosome-associated chaperone complex composed of two subunits, NACA and NACB^1,17^. Therefore, we combined all states to obtain a higher resolved reconstruction (~ 3 Å overall, Supplementary Fig. 2d-g, Supplementary Table 2) of the ribosome-bound NMT1-NAC complex (~ 4–7 Å locally, Supplementary Fig. 2h and i). Due to the high content of non-translating 80S the 3D reconstructed map resembled a SERBP1 and eEF2 bound hibernating ribosome with an E-site tRNA^18^ (Fig. 1b). In this combined dataset NMT1 and NAC were found in close proximity to each other engaged with the ribosome at the ribosomal tunnel exit. After the combined refinement of hibernating and actively translating ribosomes, we isolated an 80S class in an A/P P/E hybrid state, indicative of an actively translating 80S (Fig. 1c). Despite the comparably lower resolution of this class (~ 4.1 Å, Supplementary Fig. 2c), we could clearly observe that NMT1-NAC associated with the active ribosome in the same position found in the hibernating or combined classes, respectively (Fig. 1b, c, Supplementary Fig. 2c). Moreover, in all classes we observed NMT1 to appear always together with NAC. This indicates that for N-myristoylation of nascent peptides in vivo, NMT1 interacts with the ribosome in a specific conformation near the tunnel exit of the 60S subunit in the presence of NAC (Fig. 1b, c). This positioning near the ribosomal tunnel exit for immediate access to the emerging nascent peptide is similar to recent observations of the N-acetyl transferases NatA and NatB in yeast^19,20^, and NatA/NatE together with NAC and METAP1 in humans^21,22^. As observed also for these co-translationally acting enzymes, NMT1’s conformation is apparently not affected by the presence or absence of a nascent chain, and the affinity of NMT1 to non-translating ribosomes was sufficient to allow for efficient co-purification.

It was shown for human N-acetyltransferase NatA/NatE and the methionine excision enzyme METAP1 that the co-translational activity depends on the coordination by the NAC complex^21,22^. Since N-myristoylation by NMT1 also requires removal of the starting methionine to expose the target glycine residue^16^, we were wondering whether NMT1 binding is also dependent on NAC in a similar fashion. Therefore, we first performed an AlphaFold 2 (AF2) multimer^23,24^ analysis asking whether a potential interaction between NMT1 and NAC can be predicted. AF2 indeed predicted with confidence an interaction between the very C-terminus of NACB (140-162aa) and a cavity on the solvent exposed side of the catalytic GNAT (GCN5-related N-acetyltransferase) domain of NMT1 (Fig. 1d and Supplementary Fig. 1b, c). Interestingly, this region of NACB (Supplementary Fig. 1d, 146-VPDLV-150) overlaps with the previously reported hydrophobic motif required for METAP1 interaction^25^. In addition, it includes an acidic patch (140–145aa) and the remaining C-terminus (151-162aa) flanking the METAP1 motif (Supplementary Fig. 1d, e). Based on this prediction we aimed at testing the potential co-association of NMT1 and NAC with ribosomes and performed in vitro binding assays. We co-incubated GST-tagged NMT1, purified 80S ribosomes with the NAC dimer containing either a wildtype NACB subunit or different NACB C-terminal mutant constructs (Fig. 1f, g, Supplementary Fig. 1g, h). Consistent with what has been reported before^26^, we found that NMT1 can efficiently associate with 80S ribosomes even in the absence of NAC. While addition of the NAC complex did not increase the binding, co-association was detected with the wildtype NAC complex in roughly stochiometric amounts compared to 80S (Fig. 1g). However, the binding capacity of NAC to NMT1-ribosome complexes was strongly diminished for all NAC constructs containing truncations or substitutions in the C-terminus of NACB, yet, without influencing the ribosome binding of NMT1. Progressive truncation of NACB from the C-terminus showed that removal of the terminal 6 amino acids is already sufficient to substantially diminish NAC co-association in vitro. In addition, the sole mutation of F153 that is predicted to bind into a hydrophobic pocket of NMT1 (Supplementary Fig. 1e, lower panel) was also sufficient to perturb binding. Consistently, substitution of F153 and its immediate flanking amino acids with alanine (6xA) had the same effect. This suggests that - while binding of NMT1 to ribosomes is largely independent of NAC in vitro - multiple components of the C-terminal stretch of NACB are necessary for ribosome association of NAC when bound together with NMT1.

### Structure of reconstituted NMT1-NAC-ribosome complexes

We set out to structurally characterize defined ribosome-nascent chain complexes (RNCs) by cryo-EM after in vitro reconstitution with purified NMT1 and NAC. We, thus, prepared RNCs using a cell-free human in vitro translation system with exogenous mRNA encoding the NMT1 substrate ARF1, containing a C-terminal human cytomegalovirus (hCMV) stalling sequence that allowed us to efficiently halt translation as well as to control the nascent chain length. The original ARF1 N-terminal sequence was replaced with an optimized peptide (GNSFSKPR) preceded by a 3xFLAG tag and a TEV cleavage site. This N-terminal peptide was previously shown to have a strong affinity to NMT1^6^, allowing us to catch the transient interaction between the substrate and NMT1 at the ribosome tunnel exit. These stalled RNCs were purified via the N-terminal 3xFLAG tag of the nascent polypeptide (Supplementary Fig. 3a). TEV protease cleavage allowed for the removal of the affinity tag and simultaneously exposed the terminal glycine as neo-N-terminus of an NMT1 target sequence, thereby creating a co-translational pseudo-substrate (Supplementary Fig. 3b). These RNCs were incubated with NMT1 and wildtype NAC and subjected to cryo-EM SPA (Supplementary Fig. 4a, b). After 3D classification (Supplementary Fig. 4c) we found 80S ribosomes containing both eRF1 in the A-site and prolyl-tRNA in the P-site, in accordance with the known hCMV stalling mechanism which is based on impaired release of the nascent chain by eRF1 (Supplementary Fig. 3c)^27^. On these stalled RNCs we observed NMT1 and NAC in the same positions identified in the complexes isolated from cells (Supplementary Fig. 3d and Fig. 1b, c). Interestingly, despite NMT1’s capacity to associate with 80S ribosomes in the absence of NAC in our binding assays, we did not observe any RNCs with only NMT1 bound but instead always in the presence of the NAC complex. This confirmed that NMT1 can efficiently stabilize or recruit NAC when associated with the ribosome.

### The NMT1-NAC-ribosome interaction

**Fig. 2:**
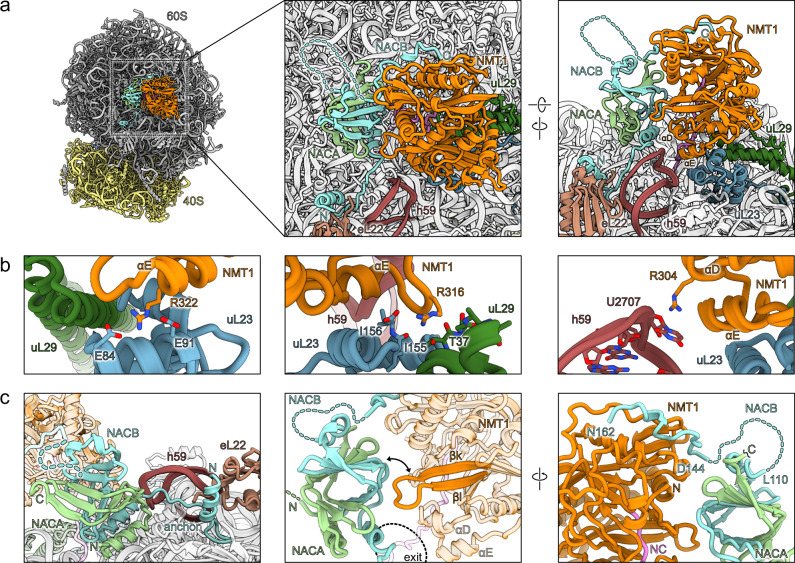
Structural details of NMT1-NAC-ribosome interaction. **a** Overview of the NMT1-NAC assembly at the peptide exit tunnel with relevant rRNA elements and ribosomal proteins highlighted. **b** Details of NMT1 ribosome anchor interactions. Prominent residues for the interaction with uL23, uL29 and 28S rRNA h59 are shown. **c** Details of the NAC complex interactions with the ribosome and NMT1. The NACB ribosome anchor (left), the NAC globular domain (middle) and the NACB C-terminus (right) interaction with NMT1 are shown.

**Fig. 3:**
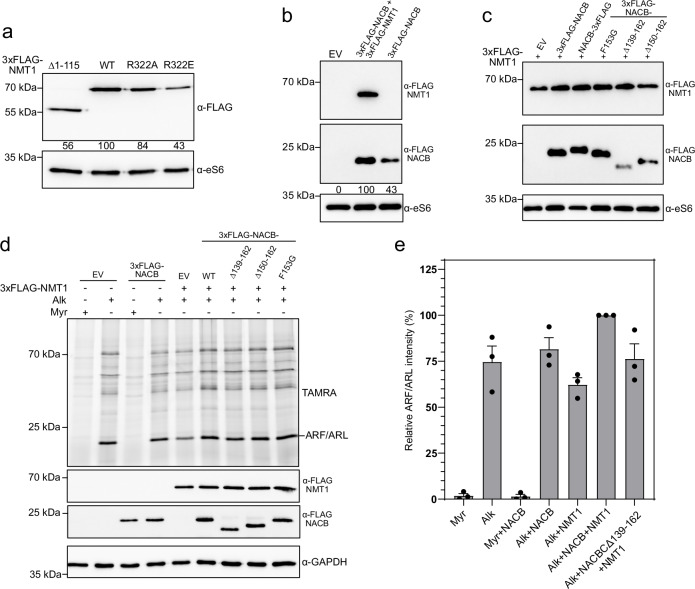
Functional analysis of NMT1–NAC interactions on ribosome–nascent chain complexes in human cells. *n* = 3 for all experiments displayed in the figure. In panels **b** and **c** only, the samples originate from the same experiment, and the gels/blots were processed in parallel, as the FLAG-NACB (~ 22 kDa) and eS6 (~28 kDa) signals fall within the same molecular-weight region of the gel. **a** HEK293 cells were transfected with plasmids encoding 3xFLAG-NMT1 and/or the 3xFLAG-NACB or the indicated variants. Ribosomal pellets were analyzed by immunoblotting with the antibodies listed to the right of each blot. Signal intensities (normalized to ribosomal protein eS6) are shown, 3xFLAG-NMT1-WT (wildtype) set to 100. R322A/E substitutions diminish NMT1 ribosome association. **b–e** HEK293 cells were cultured with myristate (Myr) or AlkC14 (Alk) and transfected with the indicated constructs encoding 3xFLAG-NMT1, 3xFLAG-NACB, or the indicated variants (EV, empty vector). Ribosomal fractions were analyzed by immunoblotting with the antibodies listed to the right of each blot. Signal intensities (normalized to ribosomal protein eS6) are shown in **b**, with 3xFLAG-NACB set to 100. Ribosomal fractions were analyzed by immunoblotting with the antibodies listed to the right of each blot. Quantification of signal intensities (normalized to ribosomal protein eS6), with 3xFLAG-NACB + 3xFLAG-NMT1 set to 100 are displayed in Supplementary Fig. 5a. Whole-cell extracts were subjected to click chemistry with a TAMRA probe and analyzed by in-gel fluorescence, with GAPDH as loading control (**d**, **e**). 3xFLAG-NMT1 and 3xFLAG-NACB expression of each lane is displayed. The corresponding Coomassie-stained gel is displayed in Extended Fig. 3b. TAMRA fluorogram with ARF/ARL band indicated (**d**). **e** Quantification of the ~20 kDa ARF/ARL signal (**e**); mean values are displayed and error bars represent the standard error of the mean; full-lane intensities is displayed in Supplementary Fig. 5c. Source data are provided as a Source Data file.

Next, we analyzed the molecular basis of the NMT1 interaction with the ribosome and with the NAC complex. NMT1 consists of two GNAT domains of which only one is catalytically active (catalytic GNAT & GNAT2)^15^. We observed the catalytic GNAT domain aligned with the peptide exit tunnel and oriented with its substrate binding cavity towards the nascent polypeptide chain (Fig. 2a). The GNAT2 domain provided the main contact sites with the ribosome (Supplementary Fig. 3e). Here, a positively charged region, encompassing two alpha helices (αD and αE) with a connecting loop (ribosome anchor region), was inserted into the negatively charged cleft between the 28S rRNA helix h59 and the ribosomal protein uL23 (Supplementary Fig. 3e, f). Due to flexibility, the overall local resolution of the NAC-NMT1 complex was lower compared to the monolithic 80S (~ 3–7 Å, Supplementary Fig. 4h, i), however, the NMT1 ribosome anchor region was well resolved (Supplementary Fig. 3g). Thus, we identified some key molecular interactions, for instance, R322 of NMT1 is in close contact with two glutamates of uL23 (E84, E91) (Fig. 2b, left), and the NMT1 loop residue R316 mediating interactions with both uL23 and uL29 via the backbone oxygens of I155, I156 and T37, respectively (Fig. 2b, middle). Further, we observed R304 interacting with the ribose oxygen of U2707 in the loop turn region of rRNA helix h59, however, given the flexibility of RNA loops possibly also allowing π-charge stacking with the base itself (Fig. 3b, right). On the other hand, the unstructured N-terminal extension of NMT1 (1–104aa) was not visible. Overall, the interaction of NMT1 with the ribosome appeared to be identical when a short substrate was provided in vitro compared to the ribosome-bound complex obtained ex vivo (Supplementary Fig. 3h).

As previously observed, the NAC complex was anchored to the ribosome via the NACB N-terminal alpha-helix hooked to ribosomal protein eL22 which tethers the globular domain of NAC (beta-sandwich) in proximity to the peptide exit tunnel, essentially as reported before (Fig. 2c, left and Supplementary Fig. 3f, g)^21,22,25^. Here, the beta-sandwich formed between NACA and NACB is positioned adjacent to NMT1 and in close proximity to a beta-turn loop (βk-βl) of the GNAT2 domain (Fig. 2c, middle). However, the low local resolution of the NAC globular domain suggests a certain degree of flexibility and instead of strongly interacting with NMT1 rather assisting the overall positioning of NMT1. Despite the lower resolution at the ribosome distal region of NMT1, we further observed additional density at the cavity on the catalytic GNAT domain that AF2 predicts as binding site for the NACB C-terminus (Fig. 2c, right). When compared to surface representations of NMT1 with and without bound NACB C-terminus at the local resolution of the map it became evident that the extra density can be best explained by the presence of the NMT1 interaction motif as predicted (Supplementary Fig. 3i). Thus, in agreement with the AF2 prediction and our binding assays, we concluded that in our complex the NACB C-terminus is indeed interacting with the crevice of NMT1 and that it contributes to the stabilization of the NMT1-NAC-ribosome complex. Interestingly, the ribosome anchor motif seems to be conserved within human NMT2 in sequence and fold (Supplementary Fig. 3j), and AF3 predicts an identical interaction with the NACB C-terminus (Supplementary Fig. 3k), hinting to a similar mode of ribosome binding.

### NAC-NMT driven myristoylation in cellulo

To further probe the binding capacity of NMT and NACB, we expressed N-terminal FLAG-tagged versions of both proteins in human cells, either individually or in combination. Consistent with our structural observations, R322 substitutions impaired NMT1 binding to ribosomes in cellulo (Fig. 3a). We also examined the unstructured N-terminal extension of NMT1, which was unresolved in our structures, and confirmed that it also contributes to ribosome binding (Fig. 3a), in agreement with earlier findings^26^.

Interestingly, NACB binding was markedly enhanced (2.3-fold) upon NMT1 overexpression, whereas the reciprocal effect was modest (+ 25%) (Fig. 3b, c). These data indicate that NMT1 binding to the ribosome occurs largely independently of NAC, while NAC recruitment is facilitated by NMT1. Supporting this view, NACB deletion variants displayed reduced ribosome binding in the presence of NMT1, whereas NMT1 binding to ribosomes was unaffected (Fig. 3c; Supplementary Fig. 5a). We next assessed the functional impact of these interactions on the overall N-myristoylation, focusing on the contribution of the NACB C-terminal domain (Fig. 3d, e). Co-expression of NMT1 and NACB altered the myristoylation profile, affecting both the major ~20 kDa substrates (accounting for ~20% of the total signal, consistent with ARF/ARL proteins) and the global myristoylome (+ 60%; Fig. 3d, e; Supplementary Fig. 5b). These findings suggest that the NACB-NMT1 interaction enhances NMT1 activity to promote optimal N-myristoylation. However, this synergistic effect does not appear to be strictly required for the reaction to occur.

Thus, in contrast to human NatA/NatE and METAP1^21,22^, which require NAC for efficient ribosome binding, NMT1 associates independently and instead stabilizes NAC on the ribosome via interaction with the NACB C-terminus. Importantly, while NMT1 does not depend on NACB for ribosome recruitment, NACB binding to NMT1 exerts a positive influence on overall protein N-myristoylation.

### NMT1 substrate interaction and MetAP coordination

**Fig. 4:**
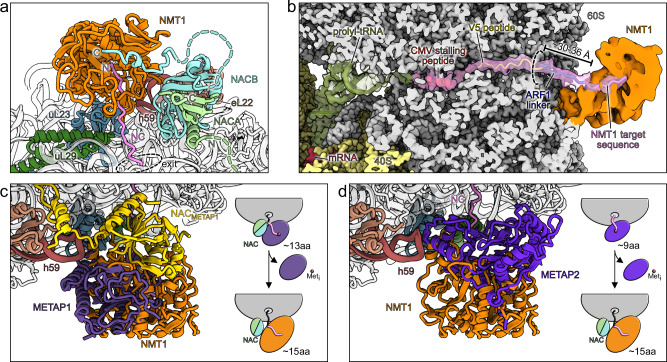
Nascent chain requirements for N-myristoylation and implications for methionine excision. **a** Overview of the NMT1-NAC assembly on the ribosome as molecular model focusing on the nascent chain (NC, pink) path from exit site to the NMT1 active site. **b** Cut-through view of the NMT1 bound RNC showing the path of the nascent polypeptide through the peptide exit tunnel towards NMT1. 80S and mRNA are shown as surface representations. Filtered density is shown for NMT1 and NAC has been omitted for clarity. Filtered density was zoned around prolyl-tRNA and the nascent chain. The molecular model for the nascent chain was colored according to the individual segments. Overlay of molecular models for ribosome bound NMT1 with either NAC-METAP1 (PDB: 8P2K) (**c**) or METAP2 (PDB: 8ONY) (**d**). NMT1 associated NAC was omitted in both cases. Cartoon representations on the right sides (**c** and **d**) showing the sequential order of events for methionine excision and myristoylation as well as estimated minimum nascent chain length requirements after exiting the peptide tunnel for activity of MetAPs and NMT1.

A fundamental prerequisite for co-translational N-myristoylation by NMT1 is prior removal of the initiator methionine, catalyzed by METAP1 or METAP2. Given that all three enzymes - METAP1, METAP2, and NMT1 - bind in proximity at the ribosomal tunnel exit, it is crucial to understand the order and coordination of ribosome binding events by these enzymes which compete for accessibility to the emerging nascent peptide. Here, due to the overall high resolution (2.19 Å) of our cryo-EM reconstruction (Supplementary Fig. 4c, e, Supplementary Table 2) we were able to trace the nascent polypeptide from the P-site tRNA until it reaches NMT1 (Fig. 4a, b). While the immediate C-terminal hCMV stalling peptide is resolved to amino acid sidechain levels for both its helical and extended part, the resolution decreased for the following V5-tag (Supplementary Fig. 3b) as the peptide exit tunnel widens and nascent chain flexibility increases. Yet, the distinct V5 sequence PIPNP is recognizable at the narrow peptide tunnel exit passage, essentially starting with the ten amino acid ARF1-derived linker (ARF1 9-18aa) after exiting the ribosome (Fig. 4b). Despite the dynamic nature of the linker, we observed density at low contour level (~ 6 Å local resolution, Supplementary Fig. 4h, i) connecting the nascent chain from the peptide tunnel exit to the substrate binding cavity of NMT1. Additional density in the known substrate binding site of NMT1 is therefore most probably representing our N-terminal pseudo target sequence (Fig. 4b). At the observed position of NMT1 we estimated that a nascent peptide needs to cover a minimal distance of ~30-36 Å to reach the N-myristoyl transferase. Including the eight amino acid substrate sequence and adjusting for the compaction by the hCMV helical fragment, NMT1 engagement could begin after at least ~45 amino acids have been translated, fully consistent with previous findings indicating less than 100 residues are required^28^. As N-myristoylation relies on the preceding excision of the initial methionine, we compared existing structural data of ribosome bound METAP1^25^ and METAP2^29^ with our data. Interestingly, binding of NMT1 to ribosomes is mutually exclusive with both MetAPs as binding sites largely overlap (Fig. 4c, d, Supplementary Fig. 6a–c). This indicates a clear sequential order of ribosome association for their consecutive co-translational modification activities, where MetAP engagement and dissociation precedes NMT1 binding to the ribosome. This differs from the concerted mode of binding suggested for METAP1 and NatA/E for methionine excision and N-terminal acetylation. There, both factors have been shown in vitro to be able to assemble at the peptide exit site coordinated by the NACB METAP1 motif and the NACA UBA domain, respectively, potentially allowing for a seamless substrate handover^21^. While co-translational activity of METAP2 is independent of the NAC complex, METAP1 relies on the hydrophobic motif in the NACB C-terminus^25^. As this motif is also part of the NACB-NMT1 interaction motif and METAP1 and NMT1 would compete for binding (see Supplementary Fig. 1e), it supports the idea of a consecutive coordination by NAC for the METAP1 and NMT1 ribosome association. In this scenario, the dissociation of METAP1 would go along with a ~ 32° rotation of the NAC globular domain away from the peptide exit, making space for NMT1 accommodation (Supplementary Fig. 6d). This conformational rearrangement highlights the structural plasticity of NAC, which can adapt its position on the ribosome depending on the specific co-translational partner recruited. Differences in ribosome engagement between METAP1 and METAP2 further suggest distinct temporal windows of action: METAP2, for instance, is positioned closer to the peptide tunnel exit, in theory requiring only a minimum of 9 amino acids to protrude from the ribosome. For METAP1, on the other hand, the active site is angled further away from the peptide exit requiring at least about 4 amino acids more, 13 altogether. Yet, NMT1 activity needs at least 15 amino acids to have left the ribosome, putting the order of events also in a plausible perspective with respect to translation progression and nascent peptide accessibility. Taken together, our structural data provides evidence for sequential co-translational methionine excision and N-myristoylation executed via consecutive binding of the respective enzymes. Several lines of evidence have shown that in eukaryotes METAP1 and METAP2 have overlapping activity, both supporting NMT1 activity^30–33^. Thus, the cell appears to maintain parallel pathways that ensure robust processing of N-terminal methionines, thereby preparing nascent substrate peptides for NMT1 modification, potentially involving a handover by NAC in case of METAP1.

### NMT1 substrate folding on the ribosome

**Fig. 5:**
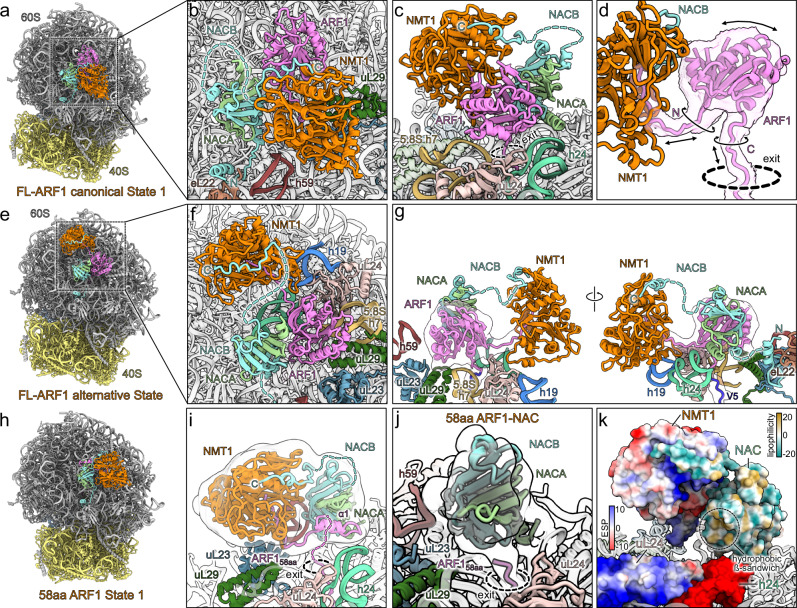
Structural details of intermediate and full length ARF1 behavior. **a** Molecular model of the canonical NMT1 State 1 highlighting the overall positioning at the peptide exit tunnel. **b** Overview of the NMT1-NAC assembly with relevant rRNA elements and ribosomal proteins highlighted. **c** Close-up of the nascent ARF1 domain wedged between the NMT1-NAC and the 60S. **d** Connection of nascent ARF1 with the polypeptide chain and NMT1. For clarity filtered, zoned density is shown. **e** Molecular model of the FL-ARF1 alternative NMT1 binding state highlighting the overall positioning at the peptide exit tunnel. **f** Overview of the alternative NMT1-NAC assembly with relevant rRNA elements and ribosomal proteins highlighted. **g** Close-up showing the orientation of the alternative ARF1 positioning and its connections with the nascent peptide and NMT1 as well as NMT1-60S interactions. For clarity filtered, zoned density is shown for ARF1. **h** Molecular model of NMT1-NAC bound 58aa-ARF1-State 1 RNCs. **i** Close-up of the nascent 58aa-ARF1 nascent peptide engaged by NMT1 and NAC in the 58aa-ARF1 State 1. Transparent filtered, zoned density for the exit site assembly is shown. **j** Close-up of peptide exit site for the NAC-bound nascent 58aa-ARF1 RNC. Transparent filtered, zoned density for NAC and the nascent peptide is shown. **k** Overview of the confined space formed by NMT1, NAC and the 60S surface. NMT1, NAC, ribosomal protein uL24 and the 28S rRNA helix h24 are shown as surface representations. NMT, uL24 and h24 are colored according to electrostatic potential (ESP) and NAC is colored according to lipophilicity.

We wondered whether NMT1 has the capacity to engage ribosomes with further elongated nascent peptides. To that end we used the same human cell-free translation system to produce RNCs similar to the one described above, however, with a full-length ARF1 substrate linker (9-181aa, including the cleavable N-terminal tag). After protease cleavage this potential substrate also contains the same glycine neo-N-terminus but remains tethered to the ribosome via linkage to the hCMV staller and the V5 tag (Supplementary Fig. 7a). These RNCs were reconstituted with NMT1 and NAC as described for the RNCs with shorter substrate and subjected to structure determination by cryo-EM SPA (Supplementary Fig. 8a, b). After 3D classification we observed a class essentially resembling the previous RNC with the short substrate, however, in addition to NMT1 and NAC we found additional density wedged between NMT1, the NAC beta-sandwich and the ribosome surface (FL-State 1) (Supplementary Fig. 8c). This additional density corresponded in shape and size to ARF1 in an almost completely folded state which allowed docking of a crystal structure of ARF1 into the density (Fig. 5a–c). Despite the limited local resolution (~ 3-9 Å for ARF1 domain, Supplementary Fig. 8h, i) for the exit site assembly, we observed clear connections to the ARF1 density from the peptide exit tunnel and the NMT1 substrate binding cavity (Fig. 5d). These pointed towards the rough orientation of the ARF1 domain and also confirmed the engagement of NMT1 with the co-translational largely folded substrate. However, the unstructured N- and C-terminal connections of the ARF1 domain allow for a certain degree of rotation and movement preventing unambiguous placement of the domain. Regardless of its dynamic nature the GTPase domain is positioned on top of ribosomal protein uL24, 28S rRNA helix 24 and 5.8S rRNA helix 7 (Fig. 5c). The ARF1 domain flexibility, however, was also reflected by our 3D classification results (Supplementary Fig. 8c) where we found a second class with a slightly displaced ARF1 (FL-State 2) (Supplementary Fig. 7b). In this state, NMT1 is tilted towards the peptide exit and the NAC globular domain shifted as well, both factors following the ARF1 domain movement (Supplementary Fig. 7c). Between the two states the NMT1 ribosome anchor remains fixed while its two GNAT domains bend in unison with the substrate domain movement. In the same fashion NMT1 is angled further away from the peptide exit site when compared to our short substrate RNC reconstruction to give space for the folded ARF1 (Supplementary Fig. 7d). Together, these findings highlight the ability of NMT1 to dynamically adapt to its substrate during extensive peptide elongation.

Comparison with the crystal structure of nucleotide-analog-bound ARF1 suggests that its C-terminal α-helix must remain partially unfolded to connect with the V5-hCMV stalling peptide still buried in the ribosomal exit tunnel (Supplementary Fig. 7e). Commonly, ARF proteins involved in membrane trafficking employ a switch mechanism that allows for myristoyl membrane insertion in a GTP bound state or membrane dissociation after nucleotide hydrolysis by tucking the myristoyl tail into a hydrophobic crevice of the ARF protein^34^. Interestingly, the C-terminal helix contributes to the formation of this cavity with three leucine residues (ARF1: L166, L170, L173 or ARF6: L162, L166, L169) (Supplementary Fig. 8f) which makes it tempting to speculate whether complete folding of ARF proteins and shielding of the hydrophobic myristoyl moiety by NMT1 might be necessary before releasing the protein.

Unexpectedly, we observed further density on the GTPase domain of ARF1 which we could not confidently identify based on our 3D reconstruction alone (Supplementary Fig. 7g, ~6-9 Å Supplementary Fig. 8h, i). It appeared most plausible that the density belongs to either NAC or NMT1, and using AF3 to identify potential interactions, positioning of the NACA UBA domain on a hydrophobic site of the ARF1 surface was predicted (Supplementary Fig. 8h). Therefore, it is possible that in a different overall orientation of ARF1 - which is well within the limits of its degree of flexibility - this interaction may occur. This might also hint towards a role of the NACA UBA domain in assisting co-translational folding in addition to its known function in factor recruitment for signal recognition particle (SRP) or NatA/E to the ribosome^21,35^.

In addition to the canonical position of NMT1 that we found in all our cryo-EM datasets - both in vitro and ex vivo - we observed NMT1 at an alternative site (alternative State) only in our full length ARF1 RNC dataset (Fig. 5e, Supplementary Fig. 8c). Here, the almost fully folded, nascent ARF1 occupies the space directly on top of the peptide exit site but facing in opposite direction compared to the canonical FL-State 1 and 2 (Fig. 5a, b, Supplementary Fig. 7b, c), simultaneously excluding NMT1 from its h59-uL23 binding site (Fig. 5f). Instead, NMT1 is attached with its ribosome anchor to a binding patch where rRNA helices h19 and h24 as well as ribosomal protein uL24 converge (Fig. 5f, g). In this position distal of the tunnel exit NMT1 activity would require the nascent chain to span a longer distance (~ 42 Å), requiring at least 20 amino acids to have left the exit tunnel (Supplementary Fig. 7i). Here, NMT1 appears very flexibly bound, likely because it lacks the positional support of the NAC globular domain, not allowing us to resolve NMT1 or its ribosome anchor to high resolution (~ 6-9 Å, Supplementary Fig. 8h, i). The NAC globular domain was also rotated by approximately 57° and in contact with the same hydrophobic site that might be occupied by the UBA domain of NAC in FL-States 1 and 2 (Supplementary Fig. 7j). This alternative state would potentially also free the space for binding of METAP1 or METAP2 (Supplementary Fig. 7k) but the competing association of the NACB C-terminus with METAP1 and NMT1 as well as the occlusion of NAC by METAP2 oppose such a simultaneous pathway. As we observed this state only in the presence of a very long substrate we could not exclude an in vitro artifact. On the other hand, these data may indicate that NMT1 indeed has the capacity to engage substrates even after extended elongation of the nascent peptide so that an entire domain has already emerged from the peptide exit tunnel. Similar observations have been made for SRP, for example, which in *E. coli* and yeast is able to engage signal sequences of nascent peptides with substantial portions already emerged from the tunnel exit^36–38^.

More interestingly, however, is the finding that the NMT1-bound substrate could be in an almost fully folded state. Thus, the function of NMT1 may not be limited to its modification activity, but it could in addition contribute chaperoning function for its substrates together with the well-known chaperone activity of NAC^39–42^. This would point to a pathway in which, in contrast to the METAP1-NatA/E situation, a primary role of NAC may not simply be the recruitment of the modifying enzyme, but also that of a chaperone acting after modification together with its potential co-chaperone NMT1 to facilitate substrate folding.

To interrogate such a potential chaperoning function of NMT1 and NAC, we isolated RNCs as before but with an intermediate ARF1 substrate (2-9aa consensus recognition sequence, 10-59aa ARF1, total 58aa) that has no intrinsically folded domain which might already be largely assembled within the in vitro translation reaction as could be the case for the full-length ARF1. Instead, the ARF1 fragment chosen should only form the first α1 helix and remain mainly unstructured according to AF3 predictions (Supplementary Fig. 9a–c). After reconstitution of these RNCs (Supplementary Fig. 9d) with NMT1 and NAC we performed cryo-EM SPA (Supplementary Fig. 10a, b) and we indeed found two states (58aa-State 1 and 2) of NMT1-NAC bound RNCs (Fig. 5h, i, Supplementary Fig. 9e, f, Supplementary Fig. 10c) that display additional density at the NAC beta-sandwich. In both cases we could dock the AF3 prediction of the α1 helix into the extra density albeit at different orientation (Fig. 5i, Supplementary Fig. 9f), which is overall consistent with the recently reported preference for NAC to associate with simple secondary structure elements of nascent peptides^43^. However, the model only serves as a placeholder due to the limited local resolution of the substrate density (~ 6-9+ Å, Supplementary Fig. 10h, i). The main difference between the two observed 58aa-States is the flexible movement of the exit site assembly. In 58aa-State 1, NMT1 was angled closer towards the ribosome surface and a clear connection to the α1 helix but not to the peptide exit was visible (Fig. 5i and Supplementary Fig. 9g) while for 58aa-State 2 NMT1 is positioned further away from the peptide exit site and the connection between the α1 helix and the emerging nascent chain is visible but not the connection towards the NMT1-bound substrate portion (Supplementary Fig. 9f, g). This loss of connectivity in both states is likely attributed to the mostly disordered nature of the 58aa ARF1 substrate (Supplementary Fig. 9c). To assess whether the additional density observed is specific for the NMT1-NAC complex, we also isolated a separate class of RNCs from the cryo-EM dataset with only NAC present (Supplementary Fig. 10c). Here, the NAC globular domain is closely positioned at the peptide exit site (Supplementary Fig. 9h), even angled closer towards it by ~20° compared to a previous NAC-RNC structure^35^ (Supplementary Fig. 9i). However, when compared to the two NMT1-NAC bound states with the 58aa-ARF1 substrate, no additional density at NAC can be observed (Fig. 5j), showing that in this case NAC alone may not be sufficient for a chaperone-like engagement with the nascent polypeptide. Rather, the additional holdase function of NMT1 is required in synergy with NAC. For both full-length and 58aa ARF1 substrates, NMT1 is not only still binding but together with NAC and the large ribosomal subunit surface appears to provide a confined environment which may serve as a folding space for its ARF1 substrate. In addition to the spatial confinement the exit site assembly together with the ribosome provides distinct surface properties that might assist folding of nascent proteins. The NAC beta-sandwich contributes a hydrophobic surface while the ribosomal protein uL24 and rRNA helix h24 respectively provide positively and negatively charged surfaces for interaction with the nascent polypeptide.

## Discussion

In this study, we provide structural insights into the co-translational N-terminal myristoylation activity of NMT1 at the human ribosome, both in vitro and ex vivo. Further, we find that NMT1 co-associates with NAC to ribosomes and identified the NACB C-terminal tail as NMT1 interaction leash. The involvement of NAC in myristoylation in addition to its known roles in co-translational acetylation, methionine excision and protein targeting to the ER^1,21^ highlights again its vital part in the coordination of nascent polypeptide targeting factors on the ribosome.

Mechanistically, our work reveals a consecutive and mutually exclusive binding of both MetAPs and NMT1 at the tunnel exit, rather than a stable multi-enzyme complex. Recent studies, largely based on in vitro data, have shown that NAC supports the assembly of a METAP1-NatA dual-enzyme complex on the ribosome, pre-positioning both enzymes for sequential initiator methionine removal and N-α-acetylation, creating a channel for N-terminal processing^21,25^. In case of NMT1 we observe a different situation: its distinct positioning only allows for a consecutive interaction mode of METAP1/2 followed by NMT1. This consecutive mode aligns with the requirement for a free N-terminal glycine for myristoylation and suggests an inherent checkpoint to ensure correct order of modifications. Interestingly, we observe that NMT1 may also adopt an alternative binding orientation on the ribosome in certain contexts, hinting at adaptable docking modes to accommodate different nascent chain lengths or sequences. Such findings underscore a remarkable flexibility and hierarchy in ribosome-associated factor recruitment whereby NAC appears to act as gatekeeper that controls factor access and promote the correct modification sequence at the right time^1^. Our structural data indicate that NMT1 operates within this coordinated framework but unlike METAP1 or SRP, contains its own ribosome-binding module and does not strictly require NAC for ribosome attachment as shown in our in vitro reconstitution assays. This suggests that NMT1 can independently dock to the tunnel exit and may even help retain NAC at the ribosome via the NACB tail interaction. Such mutual stabilization hints at a cooperation distinct from the MetAP/NatA scenario: rather than NAC simply recruiting NMT1, NMT1 and NAC likely form a composite interface that secures both factors on the ribosome. While our manuscript was under revision, two independent reports provided structural insights into NMT1 and NMT2 ribosome binding^44,45^. These studies partially overlap with our findings on the ribosomal interaction surface and NACB association, and both propose that NACB recruits NMT to the ribosome. By contrast, our in cellulo data demonstrate that NACB contributes only marginally - through C-terminal interactions and induced cooperativity - while NMT1 binding occurs largely independently. Nevertheless, our results indicate that N-myristoylation ultimately benefits from synergistic effects of the interaction between NACB and NMT.

**Fig. 6:**
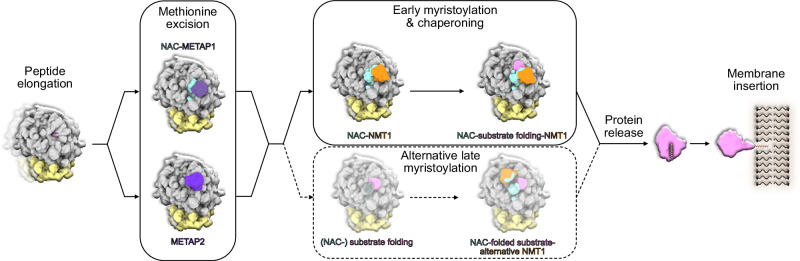
Steps of co-translational N-myristoylation by NMT1. Since the myristoyl moiety is highly hydrophobic, a partially synthesized protein with an exposed myristate could risk misfolding or aggregation. Our data support a scenario in which NMT1 is held at the ribosome until its substrate protein attains a conformation that can safely sequester or shield the lipid.

An important implication of our findings is that NMT1 may itself act as an additional co-translational chaperone-like factor in partnership with NAC - an activity not previously attributed to NMT. A long-standing question in the field is what happens to a nascent protein immediately after N-myristoylation. Since the myristoyl moiety is highly hydrophobic, a partially synthesized protein with an exposed myristate could risk misfolding or aggregation. Our data support a scenario in which NMT1 is held at the ribosome until its substrate protein attains a conformation that can safely sequester or shield the lipid (Fig. 6). Using RNCs with the GTPase ARF1, exposing the N-terminal peptide, NMT1 was still able to bind to a largely folded nascent ARF1. This is particularly significant because ARF family GTPases require complete folding to bury their myristoylated N-terminus in a hydrophobic pocket when in the GDP-bound state in the cytosol, preventing unwanted membrane attachment until the protein is activated (GTP-bound). NMT1 may act as a placeholder for shielding the myristate during translation and, in this way, the NMT1-NAC complex may cradle the nascent chain’s lipidated N-terminus to prevent premature exposure to the cytosol. Consistent with this idea, NMT1 has been reported to display slow product release kinetics and extended substrate binding periods. Indeed, the nascent chain contributes significantly to NMT binding, with an affinity in the 100 nM range^7^ which is two to three orders of magnitude higher than for other similar enzymes, such as Nats^3^ and even enhanced for the myristoylated product. For instance, the GNSFSKPR N-terminus used in this study exhibits a binding constant of 9 nM^14^. Consequently, slow product release is rate limiting in vitro^7^ and in agreement with the observation that the NMT interactome is highly enriched in its own substrates^7,46^. This further supports the model that NMT1 on the ribosome can remain bound to the nascent chain for a substantial portion of its synthesis, potentially until translation and folding are (almost) complete. We note that certain specialized chaperones for lipidated proteins mirror this principle. For example, the folding of N-myristoylated Gα subunits of heterotrimeric G-proteins (~ 355 aa) requires the dedicated chaperone Ric-8A, which binds partially folded Gα, keeps the the N-myristate solvent-protected and only releases it upon client activation (via nucleotide binding)^47,48^. Analogously, NMT1-NAC could represent an early co-translational chaperone system for nascent myristoylated proteins, shielding their hydrophobic N-terminus during biosynthesis. This function would be especially critical for large clients that also depend on auxiliary folding factors.

Our findings also emphasize the plasticity and robustness of the ribosome-associated protein biogenesis machinery. Eukaryotes express two MetAP isoforms that can compensate for each other. Both enzymes can apparently feed into the NMT1 pathway with METAP2 being NAC independent, therefore possibly benefitting from the NAC recruitment activity of NMT1 in the next step. Similarly, humans have two NMT isoforms, and while NMT1 is generally dominant and essential for cell viability, the highly conserved NMT2 can partially overlap in function (Supplementary Fig. 3k)^45^. Both MetAPs and NMTs have been pursued as drug targets in cancer, infectious disease, and other conditions. Our findings suggest that disrupting the co-translational orchestration might be as impactful as inhibiting the enzymes’ active sites. For instance, selectively targeting the NMT1-NAC interaction surface or the NMT1 ribosomal anchor might impair myristoylation of a subset of proteins with less systemic toxicity than broad active-site inhibitors. This work thus provides a framework for understanding N-terminal lipidation in the broader context of co-translational protein maturation and disease.

## Methods

### Cell lines and cell culture

Expi293F suspension cells (Thermo Fisher Scientific; #A14527) were cultured in HEK TF medium (Sartorius) supplemented with 7 mM GlutaMAX (Gibco) at densities between 0.5–4.0 × 10^6^ cells/mL at 37 °C and 5% CO_2_. HeLa S3 suspension cells (Sigma Aldrich; #87110901) were cultured in S-MEM (Gibco) supplemented with 1x GlutaMAX (Gibco), 1x Penicillin-Streptomycin (Gibco) and 10% FBS (Gibco) at 37 °C and 5% CO_2_ between 0.2 and 0.7 × 10^6^ cells/mL. Both Expi293F and HeLa S3 suspension cells were cultured at 80% humidity in a Multitron Cell (Infors HT). Adherent HEK 293T cells (ATCC; #CRL-3216) were cultured in DMEM (Gibco) supplemented with 1x GlutaMAX (Gibco), 1x Penicillin-Streptomycin (Gibco) and 10% FBS (Gibco) at 37 °C and 5% CO_2_ to maximal 80% confluency.

### Native NMT1-ribosome complex isolation

A plasmid for transient expression of NMT1 was generated by inserting the coding sequence of NMT1 which was amplified from cDNA into a pcDNA™5/FRT/TO mammalian expression vector (Thermo Fisher Scientific) that was modified to contain a N-terminal 3xFLAG-3C cleavage site tag via restriction cloning. The resulting plasmid is referred to as pMTD1-NMT1. Expi293F cells were transiently transfected with pMTD1-NMT1 using PEI at a cell density of 1.5 × 10^6^ cell/mL for 24 h. Then, cells were harvested by centrifugation at 200 x* g* for 5 min and washed once with 1x PBS. Cell pellets were resuspended in Lysis Buffer (20 mM HEPES/KOH pH 7.5, 150 mM KOAc, 5 mM MgCl_2_, 1 mM DTT, 0.5% IGEPAL CA-630 (Sigma Aldrich), protease-inhibitor mix (homemade)) and lyzed in a dounce homogenizer (Wheaton) with ten strokes using the loose pestle. The lysate was cleared by two consecutive centrifugation steps at 2960 × *g*  for 15 min and at 36,500 x* g* for 25 min. The cleared lysate was incubated with anti-FLAG M2 affinity gel (Sigma-Aldrich) for 1 h. Beads were collected by centrifugation at 600 x *g* for 3 min and the flow-through was removed. The beads were washed twice with NP-40 Wash Buffer (20 mM HEPES/KOH pH 7.5, 150 mM KOAc, 5 mM MgCl_2_, 1 mM DTT, 0.1% IGEPAL CA-630) and once with Nikkol Buffer (20 mM HEPES/KOH pH 7.5, 150 mM KOAc, 5 mM MgCl_2_, 1 mM DTT, 0.05% octaethylene glycol monododecyl ether). After transferring the beads to a 1 mL spin-column (MoBiTec) the beads were washed once more with Nikkol Buffer. NMT1-ribosome complexes were eluted in Nikkol Buffer supplemented with 0.25 mg/mL 3C protease (homemade) for 1.5 h at 4 °C, collected by centrifugation and analyzed by SDS-PAGE. For cryo-EM, eluates were crosslinked with 0.02% glutaraldehyde for 20 min on ice and quenched with 25 mM Tris.

### Sucrose density gradient analysis

After HEK 293T cells (ATCC; #CRL-3216) were grown to 40% confluency they were transfected with plasmid pMTD1-NMT1 (see above) for 24 h using PEI. Then they were treated with 100 µg/mL cycloheximide for 10 min and collected from plates by scrapping followed by centrifugation at 200 x* g* for 5 min. The cell pellet was washed once with 1x PBS supplemented with 100 µg/mL cycloheximide before resuspending it in Lysis Buffer (20 mM HEPES/KOH pH 7.5, 150 mM KOAc, 5 mM MgCl_2_, 1 mM DTT, 0.5% IGEPAL CA-630 (Sigma-Aldrich), protease-inhibitor mix (homemade), 100 µg/mL cycloheximide, 2 U/mL SUPERase·In RNase Inhibitor (Thermo Fisher Scientific)). After passing the cell suspension five times through a 26 gauge needle the lysate was cleared by centrifugation at 15,000 x* g* for 15 min. Lysate correlating to 6 A_260_ units was separated through a linear 10–50% sucrose gradient in Gradient Buffer (20 mM HEPES/KOH pH 7.5, 150 mM KOAc, 5 mM MgCl_2_, 1 mM DTT, 100 µg/mL cycloheximide, 2 U/mL SUPERase·In RNase Inhibitor (Thermo Fisher Scientific)) in an SW 40 Ti rotor (Beckman Coulter) at 28,857 x* g* for 17.5 h. Gradients were fractioned using a Biocomp Gradient Station with a TriaX Flow Cell for A_260_ measurement. Fractions were TCA precipitated and equal volumes were used for western blot detection of 3xFLAG-3C-NMT1 using a monoclonal mouse anti-FLAG HRP-conjugated antibody (Sigma Aldrich; #A8592; clone M2; lot 0000259468) diluted 1:10,000.

### Ribosomal fraction preparation

NACB and NMT1 DNA constructs were cloned into the pcDNA™5/FRT/TO mammalian expression vector (Thermo Fisher Scientific), each fused to an N-terminal 3×FLAG epitope. NMT1 variants were derived from pMTD1-NMT1, whereas NAC variants were subcloned from their pET28a derivatives (see below). HEK293 cells were cultured in DMEM (Gibco) supplemented with 10% fetal bovine serum (Gibco) at 37 °C with 5% CO₂ until reaching ~70% confluency (48–72 h) in 10-cm Petri dishes (8 mL medium). Cells were then transferred to reduced-serum Opti-MEM (Gibco) and transfected with Lipofectamine 3000 (Invitrogen) at a ratio of 1.5 µL per µg of plasmid DNA for 3 h. Co-transfections were performed with NACB and NMT1 derivatives (7 µg each), using the empty pcDNA™5/FRT/TO vector (EV) as a control. The medium was replaced with fresh DMEM + 10% fetal bovine serum. Cells were incubated for an additional 18 h before being washed with 1x PBS, dissociated using 1 mL of 0.05% trypsin-EDTA (Gibco) for 2 min at 37 °C, and collected by centrifugation at 4500 × *g* for 4 min at 4 °C. The resulting pellet was washed twice with PBS and stored at −80 °C. For lysis, cell pellets were resuspended in 1 mL of buffer A (20 mM HEPES/KOH pH 7.5, 150 mM KOAc, 10 mM MgCl₂, 1 mM DTT, and cOmplete Protease Inhibitor [1 tablet/50 mL, Roche]), homogenized using a 7-mL Dounce glass homogenizer (B pestle, 20 strokes), and centrifuged twice at 12,000 × *g* for 15 min at 4 °C. The resulting supernatant was layered over a 1-mL sucrose cushion (20 mM HEPES/KOH pH 7.5, 150 mM KCl, 10 mM MgCl₂, 1 M sucrose) in a 3.5-mL thick-wall polypropylene centrifuge tube (Beckman, #349623) and centrifuged at 250,000 × *g* for 4 h at 4 °C in a Beckman TL100 ultracentrifuge (TLA 100.3 rotor). The pellet was resuspended in 50 µL of buffer A, and protein concentration was determined using the Bradford protein assay (Bio-Rad). For immunoblotting, 10 µg of total protein per sample was separated by 12% SDS-PAGE, transferred onto PVDF membranes (Merck), and probed with either a FLAG-epitope recombinant antibody conjugated to horseradish peroxidase (anti-DYKDDDDK-HRP; Miltenyi Biotec; #130-101-572; clone REA216; lot 5160209288), anti-GAPDH (ProteinTech; #10494-1-AP; lot 00055216) or monoclonal anti-RPS6 (eS6) rabbit antibodies (Cell Signaling Technology; clone 5G10; #2217; lot 13), diluted 1:1,000 (FLAG/RPS6) or 1:10,000 (GAPDH). If needed, a secondary anti-rabbit HRP-conjugated goat antibody (Sigma Aldrich; #A0545; lot 069M4835V) diluted 1:10,000 was used for chemiluminescent detection. Detection was performed using the ECL kit (Cytiva) and visualized with a ChemiDoc imager (Bio-Rad).

### Click-chemistry

Cells were transfected as indicated before and grown thereafter in the DMEM medium supplemented with either 50 µM of 13-tetradecynoic acid (AlkC14; Activate Scientific; AS29577) or myristate (Sigma Aldrich; M8005) for click-chemistry investigation. Protein extracts from cell lysates (40 µg) were prepared, clicked for 1 h at room temperature in the presence of 1 mM CuSO_4_, 1 mM tris(2-carboxyethyl)phosphine, 100 µM tris(3-hydroxypropyltriazolylmethyl)amine and 100 µM TAMRA-azide probe. The reaction was stopped by adding 5 mM EDTA. Samples (20 µg) were resolved by SDS–PAGE (12%) and imaged with Cy3 filters on a Typhoon scanner (RGB Biomolecular Imager, GE Healthcare)^49^. The PageRuler Plus Prestained Protein Ladder was used as molecular weight marker (Thermo Fisher Scientific).

### Densitometric analysis of fluorograms

Fluorograms obtained from immunoblotting were quantified using ImageJ (NIH) on.jpg files, whereas click-chemistry analyses were performed on.tiff image files generated by the Typhoon Biomolecular Imager using Image Lab software (Bio-Rad). For ImageJ analyses, images were converted to 8-bit grayscale and regions of interest of equal size were manually drawn around each band. Background intensity was measured in an empty region of the blot and subtracted from each value. Corrected band intensities were calculated as Integrated Density (Area × Mean Gray of background). For Image Lab analyses, band detection and background subtraction were performed automatically by the software according to the default quantification settings. For ribosome fractions, target bands were normalized to the intensity of the corresponding RPS6 (eS6) band in the same lane, whereas for whole-cell extracts, normalization was performed against the GAPDH band. The same sample was measured repeatedly and error bars represent the standard error of the mean (SEM).

### In vitro transcription

A plasmid carrying a 3xFLAG-TEV-NMT1 target sequence-full length ARF1-V5-hCMV staller construct (Supplementary Fig. 1h) flanked by *X. laevis* beta globin 5’ and 3’ UTRs as well as a Kozak sequence under a T7 promoter were assembled by GeneArt (Thermo Fisher). The plasmid carrying the short ARF1-linker (9-18aa) was generated by mutagenesis PCR. DNA templates for in vitro transcription were PCR amplified from these plasmids. The resulting linear DNA fragments were used to generate capped hCMV stalling mRNAs with the mMessage mMachine T7 Transcription Kit (Thermo Fisher) according to the manufacturer’s instructions. Remaining template DNA was removed by treatment with TURBO DNase (Thermo Fisher) for 15 min at 37 °C before isolating the mRNA via LiCl precipitation.

### Human in vitro translation extract preparation

Human in vitro translation extract was prepared similarly to what was previously reported^50^. HeLa S3 cells were grown to a density of 0.6 × 10^6^ cells/mL and treated with 200 nM ISRIB for 1 h. All following steps were carried out at 4 °C or on ice. Cells were harvested by centrifugation at 650 x* g* for 7 min, and washed twice with Wash Buffer (35 mM HEPES/NaOH pH 7.5, 140 mM NaCl, 11 mM glucose) and once with Extraction Buffer (20 mM HEPES/KOH pH 7.5, 45 mM KOAc, 45 mM KCl, 1.8 mM Mg(OAc)_2_, 1 mM DTT). The cell pellet was resuspended in Extraction Buffer to 1.2 ×10^9^ cells/mL and lyzed by nitrogen cavitation in a Parr Instrument at 300 psi for 30 min. Equal to 1/29 of total lysate volume High Potassium Buffer (20 mM HEPES/KOH pH 7.5, 945 mM KOAc, 945 mM KCl, 1.8 mM Mg(OAc)_2_, 1 mM DTT) was added and the lysate was incubate for 5 min. The lysate was cleared by two subsequent centrifugation steps in a TLA-110 rotor (Beckman Coulter) at 15,700 ×* g* for 13 min and at 74,300 ×* g* for 19 min. Endogenous mRNA was removed by treatment with 75 U/mL S7 MNase and 1 mM CaCl_2_ at 23 °C for 5 min, followed by nuclease inactivation with 2.4 mM EGTA. The translation competent extract was aliquoted, frozen in liquid nitrogen and stored at -80 °C.

### Human in vitro translation

In vitro translation reactions were essentially performed as described before^50^. In brief, translation reactions were adjusted to 50%-v/v human cell extract, 20 mM HEPES pH 7.5, 0.42 mM MgCl_2_, 2.75 mM Mg(OAc)_2_, 75 mM KOAc, 37.5 mM KCl, 1.56 mM GTP, 0.25 mM ATP, 1.6 mM creatine phosphate, 50 µg/mL bovine liver tRNA (Sigma Aldrich), 0.45 mg/mL creatine kinase, 0.4 mM spermidine, 0.12 mM amino acid mix (homemade) and 0.8 U/µL SUPERaseIn RNase Inhibitor (Thermo Fisher), 1 mM DTT and 8.15 nM CMV staller mRNA. Reactions were incubated for 40 min at 30 °C before RNCs were isolated.

### RNC isolation

Ribosome species were directly pelleted from in vitro translation reactions through a sucrose cushion in AP Buffer (20 mM HEPES/KOH pH 7.5, 150 mM KOAc, 5 mM MgCl_2_, 1 mM DTT, 2 U/mL SUPERase·In RNase Inhibitor (Thermo Fisher), 0.05% octaethylene glycol monododecyl ether, 1 M sucrose) at 408,800 x* g* for 1 h using a TLA-110 rotor (Beckman Coulter). The ribosome pellet was resuspended in APS-HS Buffer (20 mM HEPES/KOH pH 7.5, 300 mM KOAc, 5 mM MgCl_2_, 1 mM DTT, 2 U/mL SUPERase·In RNase Inhibitor (Thermo Fisher), 0.05% octaethylene glycol monododecyl ether, 100 mM sucrose) and potential aggregates were removed by centrifugation at 14,000 x* g* for 15 min. The resuspension was incubated with anti-FLAG M2 affinity gel (Sigma-Aldrich) pre-equilibrated in APS-HS Buffer for 1 h at 4 °C in a 1 mL spin column (MoBiTec). Then, the beads were washed two times with APS-HS Buffer and subsequently two times with APS Buffer (20 mM HEPES/KOH pH 7.5, 150 mM KOAc, 5 mM MgCl_2_, 1 mM DTT, 2 U/mL SUPERase·In RNase Inhibitor (Thermo Fisher), 0.05% octaethylene glycol monododecyl ether, 100 mM sucrose) by gravity-flow. RNCs were eluted by TEV cleavage - revealing the NMT1 target sequence - in APS Buffer supplemented with 0.5 mg/mL TEV protease (homemade) for 1.5 h at 4 °C and collected by centrifugation. To remove TEV protease RNCs were pelleted through a 1 M sucrose cushion in APS Buffer at 358,400 x* g* for 1 h using a TLA-120.2 rotor (Beckmann Coulter) and the resulting RNC pellet was resuspended in APS Buffer. Peptidyl-tRNA integrity was assessed by Western blot using a monoclonal mouse α-V5 antibody (abcam; #ab2767; clone SV5-Pk1; lot 1025365-10) at a 1:2,000 dilution and an HRP-conjugated goat anti-mouse IgG (dianova; #115-035-003; lot 158670) at a 1:10,000 dilution as primary and secondary antibody, respectively.

### Factor-free 80S preparation

Expi293F cells were grown to 3 × 10^6^ cells/mL, harvested by centrifugation at 200  x* g* for 5 min and washed once with 1x PBS. Cells were resuspended in HS Lysis buffer (20 mM HEPES/KOH pH 7.5, 500 mM KOAc, 2 mM MgCl_2_, 1 mM DTT, 0.5% IGEPAL CA-630) and incubated for 10 min under constant agitation. The lysate was cleared by centrifugation at 2960 x* g* for 15 min and at 36,500 x* g* for 25 min. Then, ribosomes were pelleted through a 1 M sucrose cushion in HS Buffer (20 mM HEPES/KOH pH 7.5, 500 mM KOAc, 2 mM MgCl_2_, 1 mM DTT) in a Type 70 Ti rotor (Beckman Coulter) at 106,700 x* g* for 18.5 h. The ribosome pellet was resuspended in HS Buffer and nascent chains were released by incubation with 1 mM puromycin for 15 min ice followed by 10 min at 37 °C. Resulting 40S and 60S subunits were separated over 10–40% linear sucrose gradient in HS Buffer in a SW 40 Ti rotor (Beckman Coulter) at 202,496 x* g* for 2.5 h. The respective peak fractions were collected and subunits were pelleted through a 1 M sucrose cushion in LS Buffer (20 mM HEPES/KOH pH 7.5, 100 mM KOAc, 10 mM MgCl_2_, 1 mM DTT) in a Type 70 Ti rotor at 140,900 x* g* for 14 h. Subunits were resuspended in LS Buffer and potential aggregates were removed at 10,000 x* g* for 5 min. 80S ribosomes were reconstituted by incubating 40S and 60S subunits at equimolar amounts in LS Buffer for 1 h on ice. Remaining subunits were removed by separation through a 10–30% linear sucrose gradient in LS Buffer in a SW 40 Ti rotor at 202,496 x* g* for 2.5 h. The newly formed 80S were collected and pelleted through a 1 M sucrose cushion in LS Buffer in a TLA-110 rotor (Beckman Coulter) at 494,648 x* g* for 1 h. The ribosome pellet was resuspended in LS Buffer and any aggregates were removed at 10,000 x* g* for 5 min.

### GST-NMT1 purification

The NMT1 gene was subcloned from pMTD1-NMT1 into pGEX-6P by restriction cloning. *E. coli* Rosetta 2 (DE3) transformed with plasmid pGEX-6P-NMT1 were grown at 37 °C to 0.7 OD_600_ before protein expression was induced with 0.1 mM IPTG for 18 h at 18 °C. Cells were harvested by centrifugation and washed once with 1x PBS. Cell pellets were resuspended in Lysis Buffer (25 mM HEPES/NaOH pH 7.5, 500 mM NaCl, 5% glycerol, 1 mM DTT) supplemented with 0.02 mg/mL DNase I and lyzed using a continuous flow cell disrupter (Constant Systems). The lysate was cleared by centrifugation at 2960 x* g* for 15 min and at 36,500 x* g* for 25 min. The cleared lysate was incubated with Glutathione Sepharose 4 Fast Flow resin (Cytiva) for 1 h at 4 °C. Beads were collected by centrifugation at 600 x* g* for 3 min and the flow-through removed, then washed three times with Lysis Buffer. The resin was transferred to a gravity-flow column and washed twice with Lysis Buffer. GST-NMT1 was eluted in GST-Elution Buffer (50 mM Tris/HCl pH 8.0, 100 mM NaCl, 5% glycerol, 20 mM reduced glutathione, 1 mM DTT) for 1 h at 4 °C. The elution was dialyzed overnight against LS Buffer (25 mM HEPES/NaOH pH 7.5, 100 mM NaCl, 5% glycerol, 1 mM DTT) before loading onto a HiTrap SP HP column (GE Healthcare) equilibrated with LS Buffer. Impurities were removed by increasing the concentration of HS Buffer (25 mM HEPES/NaOH pH 7.5, 1000 mM NaCl, 5% glycerol, 1 mM DTT) to 15%. Subsequently, protein was eluted by linearly increasing the concentration of HS Buffer to 50%. The fractions collected containing GST-NMT1 were pooled and dialyzed overnight against LS Buffer. Dialyzed protein was loaded onto a HiTrap Q HP column (GE Healthcare) equilibrated with LS Buffer and eluted with a linear gradient up to 50% HS Buffer. Pooled fractions containing GST-NMT1 were concentrated using an Amicon Ultra Centrifugal Filter (30 kDa M_w_ cutoff, Merck) and buffer was exchanged to Storage Buffer (20 mM HEPES/KOH pH 7.5, 100 mM KOAc, 5 mM MgCl_2_, 1 mM DTT) using a Superdex S200 Increase 10/300 GL column (GE Healthcare). Finally, GST-NTM1 was concentrated as before and the final concentration was estimated by comparison with BSA using SDS-PAGE.

### NAC wildtype and mutant purifications

The gene coding for human NACA was cloned into pET28a (Novagen) using NdeI and BamHI resulting in a N-terminally His_6_-tagged NACA. In a second step the gene coding for human NACB isoform 2 preceded by a ribosome binding site was cloned into the same vector using EcoRI and NotI. The resulting plasmid coding for the human NAC heterodimer was freshly transformed into *E. coli* ER2566 (NEB) which were grown in LB medium at 37 °C to an OD_600_ of 0.8, induced by addition of 1 mM IPTG and continued to grow for 3 h. Cells were lysed using a cell disruptor (Constant Systems Ltd.) at 30 Kpsi in lysis buffer (20 mM HEPES pH 8.0, 500 mM NaCl, 0.1 mM PMSF, 20 µg/ml DNaseI and 1x cComplete EDTA-free protease inhibitor cocktail (Roche)). Lysates were clarified by centrifugation in a A27-8×50 rotor (Thermo Fisher Scientific) in a LYNX6000 centrifuge (Sorvall) at 36,500 x *g* for 25 min. The supernatant was incubated with Protino Ni-NTA Agarose slurry (Macherey & Nagel) equilibrated in lysis buffer for 30 min at +4 °C. Beads were transferred to a gravity flow column, washed with 40 column volumes of lysis buffer and eluted with 5 column volumes of lysis buffer containing 500 mM imidazole. NAC containing fractions were pooled and dialyzed (M_w_ cutoff 12–14 kDa) over night against buffer B (20 mM HEPES pH 8.0, 50 mM NaCl, 2 mM CaCl_2_) in the presence of thrombin (Merck) to remove the His_6_-tag. Dialyzed samples were loaded on a HiTrap SP HP column (Cytiva) pre-equilibrated with 20 mM HEPES pH 7.0, 50 mM NaCl for removal of excess NACA and eluted with a 6-column volume linear gradient to 100 % Buffer C (20 mM HEPES pH 7.0, 1 M NaCl). Heterodimeric NAC eluted at around 450 mM NaCl. PD10 columns (Cytivia) were used to transfer heterodimeric NAC fractions into the final Buffer D (20 mM HEPES pH 7.5, 100 mM KOAc, 5 mM MgCl_2_, 5 % glycerol and 1 mM DTT). Protein concentrations were determined using the molar extinction coefficient of human NAC (ε280 = 2,980 M^−1^ cm^−1^) and estimated by comparison with BSA using SDS-PAGE. All NAC mutants were expressed and purified the same way with the exception of the HiTrap SP HP column (Cytiva) which was performed at pH 8.0 for all mutants.

### In vitro binding assay

3 pmol factor-free 80S with 24 pmol GST-NMT1 and 24 pmol NAC-wt, -mutants or individual components were incubated in Binding Buffer (20 mM HEPES/KOH pH 7.5, 100 mM KOAc, 5 mM MgCl_2_, 2 mM DTT) for 1 h on ice. Mixtures were incubated with Glutathione Sepharose 4 Fast Flow resin (Cytiva) pre-equilibrated in Binding Buffer for 1 h at 4 °C with overhead rotation in a 1 mL spin- column (MoBiTec). The flow-through was collected by centrifugation at 450 x* g* for 10 s and the beads were washed three times with Binding Buffer. Bound factors were eluted by incubation with Binding Buffer supplemented with 25 mM reduced glutathione for 1 h at 4 °C. Elution fractions were TCA precipitated and equal volumes were analyzed on 12% NuPAGE gels (Invitrogen). Elution fractions were also probed by immunoblotting using a polyclonal rabbit α-NACA^17^ antibody diluted 1:2000 and monoclonal rabbit α-eS10 (abcam; #ab151550; clone EPR8545; lot GR3396422-8) antibody diluted 1:1,000. HRP-conjugated goat anti-rabbit IgG (Sigma Aldrich; #A6154; lot SLBG7201V) was used as secondary antibody at a 1:10,000 dilution.

### RNC-NAC-NMT1 in vitro reconstitution for cryo-EM

100 nM RNCs were incubated with 135 nM wildtype NAC and 135 nM GST-NMT1 in Reconstitution Buffer (20 mM HEPES/KOH pH 7.5, 150 mM KOAc, 5 mM MgCl_2_, 1 mM DTT, 0.05% octaethylene glycol monododecyl ether, 100 mM sucrose) for 1 h on ice before directly preparing cryo-EM grids.

### Cryo-EM grid preparation

For both native and in vitro reconstituted samples 3.5 µL were applied to R3/3 300 mesh carbon support copper grids with 3 nm continuous carbon (Quantifoil) using a Vitrobot (FEI) at 5 °C and 85% humidity. Grids were blotted for 3 s with 45 s pre-blotting time before plunge freezing in liquid ethane.

### Cryo-EM data acquisition and processing

Cryo-EM data for the native NMT1 sample were collected on a Titan Krios (FEI) at 300 kV equipped with a K2 summit direct electron detector (Gatan) in counting mode at a nominal pixel size of 1.049 Å/pixel, a total dose of 45.6 e^-^/Å^2^ and a defocus range from −0.5 to −3.5 µm. For the in vitro reconstituted short (10aa) ARF1-linker, 58 amino acid ARF1-linker and full length ARF1 RNCs with NMT1 and NAC samples, data were collected with a Titan Krios (FEI) with a Falcon 4i detector (Thermo Fisher) and a Selectris X energy filter (Thermo Fisher) at 300 kV and 5 eV slit range using EPU v3.3.1 (Thermo Fisher Scientific). A nominal pixel size of 0.727 Å/pixel, a defocus range from −0.5 to −3.5 µm and a total dose of 40 e^-^/Å^2^ were used. For all data collections movies were motion corrected using MotionCor2^51^ and contrast-transfer function (CTF) parameters were estimated using CTFFIND4^52^.

For the native co-IP sample, micrographs with a resolution above 5 Å were sorted out resulting in a total of 6,552 images (Supplementary Fig. 2a). Particles were picked on low-pass filtered micrographs using the general model of crYOLO^53^ (v1.7.6) and extracted three times binned with 150 pixels boxsize using Relion^54^ (v4.0.1). Extracted particles were imported to cryoSPARC^55^ (v4.4.0) and subjected to 2D classification. 215,295 particles corresponding to 80S ribosomes were selected (Supplementary Fig. 2b) and an ab initio volume was generated which was used as reference for a Homogenous Refinement (Supplementary Fig. 2c). To sort for different translational states alignment-free 3D classification was used yielding various classes consisting of hibernating as well as active 80S and bad particles (6%). After 3D classification all good classes already show clear density for NAC-NMT1. Therefore, particles from a class containing hybrid (A/P P/E tRNA) ribosomes - representative for co-translationally bound NAC-NMT1 - (4%; 7,959 particles) were selected and re-extracted in Relion with 450 pixels boxsize and without binning. They were re-imported into cryoSPARC and refined using Homogeneous Refinement followed by local resolution estimation and filtering (Supplementary Fig. 2c). However, since the majority of 80S particles were hibernating ribosomes, all good 80S classes from the initial classification (94%) were combined to allow for a higher resolution reconstruction. These classes were refined together and subjected to focused 3D classification in cryoSPARC using a mask on the exit tunnel site (Supplementary Fig. 2c). The three resulting classes were either EBP1, NAC or NAC-NMT1 bound 80S. NAC-NMT1 bound 80S particles (28%; 60,808 particles) were re-extracted in Relion with 450 pixels box size and without binning. They were refined to an overall resolution of 2.95 Å in cryoSPARC using Homogenous Refinement with global CTF and per particle defocus refinement (Supplementary Fig. 2d-g, Supplementary Table 2).

For the short (10aa) ARF1-linker RNC-NMT1-NAC sample, a total of 38,099 micrographs were collected (Supplementary Fig. 4a). Particles were picked with crYOLO^53^ (v1.7.6) from lowpass filtered micrographs using the general model and extracted four times binned in Relion^54^ (v4.01) with a box size of 160 pixels. Extracted particles were subjected to 2D classification in cryoSPARC^55^ (v4.6.0). Good 80S classes (Supplementary Fig. 4b) amounting for 788,761 particles were selected and used for homogenous refinement with an ab initio generated reference. Classes containing P-site tRNA and eRF1 were selected from alignment-free 3D classification in cryoSPARC as representatives for confident stop codon stalling by the hCMV peptide (43%; 341,641 particles) (Supplementary Fig. 4c). Stalled RNCs were further 3D classified using a spherical mask around the peptide exit site which - after removal of particles with bad exit density - yielded 163,265 final particles (21%). These were re-extract un-binned in Relion with a boxsize of 640 pixels and refined in cryoSPARC to an overall resolution of 2.19 Å using non-uniform refinement with a dynamic masking start resolution of 1 Å, global CTF and per particle defocus refinement (Supplementary Fig. 4d–g, Supplementary Table 2).

For the full length ARF1 RNC-NMT1-NAC sample, a total of 57,603 micrographs were collected (Supplementary Fig. 8a, c). Particles were picked using the general model from crYOLO^53^ (v1.7.6) on low pass filtered micrographs and extracted with Relion^54^ (v4.0.1) four times binned with a box size of 160 pixels. Extracted particles were imported in cryoSPARC^55^ (v4.6.0) and subjected to 2D classification. 1,115,225 particles were selected corresponding to 80S ribosomes classes (Supplementary Fig. 8b). An ab initio reference was generated and used for the initial 3D homogenous refinement. Particles were then sorted by alignment-free 3D classification in cryoSPARC. The class containing eRF1 as a representative for confident hCMV stalling was selected (30%; 331,442 particles) and imported back to Relion for further classification. First a wide mask covering the peptide exit tunnel site was used for focused 3D classification, resulting in one class containing NMT1 and NAC in its canonical positions (8%) and one where NMT1 and NAC were observed in different positions (8%). In both cases additional density corresponding to the ARF1 domain was observed. The canonical NMT1-NAC class was further classified with a mask around ARF1 revealing two states with different NMT1-NAC-ARF1 conformation termed State 1 (6%; 62,249 particles) and State 2 (2%, 21,642 particles). The alternative NMT1-NAC-ARF1 particles were further classified with a mask around the alternative NMT1 position, yielding a class with reasonable density (4%; 44,109 particles) and one with diffuse density. Particles for the canonical NMT1 states (State 1 and 2) and for the alternative state were re-extracted un-binned with 640 pixels box size. Finally, they were refined in cryoSPARC using Homogenous Refinement with global CTF and per-particle defocus refinement leading to overall resolutions of 2.47 Å (State 1), 2.75 Å (State 2) and 2.57 Å (alternative NMT1 state) (Extendad Data Fig. 8c-g, Supplementary Table 2).

For the 58 amino acid ARF1-linker RNC-NMT1-NAC sample 46,292 micrographs were collected (Supplementary Fig. 10a). Particles were picked with crYOLO (v1.7.6) using the general model on low pass filtered micrographs. They were extracted in Relion (v4.0.1) four times binned with a box size of 160 pixels and subjected to 2D classification in cryoSPARC (v4.6.0). A total of 1,008,730 particles corresponding to 80S ribosomes were selected (Supplementary Fig. 10b) and refined using homogeneous refinement with an ab initio generated reference volume. Then particles were subjected to alignment-free 3D classification in cryoSPARC and the class containing eRF1 was selected (30%; 299,371 particles, Supplementary Fig. 10c). Particles were imported back to Relion and 3D classified using a wide mask around the peptide exit site. Two classes containing NMT1 and NAC with pronounced nascent chain density outside the peptide exit tunnel, but different conformations (58aa-State 1: 6%; 60,443 particles and 58aa-State 2: 5%; 49,106 particles) of the exit site assembly as well as a class containing only NAC (8%; 74,754 particles) where selected (Supplementary Fig 10c). They were refined in Relion and re-extracted un-binned with a box size of 640 pixels. Particles for each class were then imported to cryoSPARC and refined using Non-uniform refinement with global CTF and per particle defocus refinements resulting in overall resolution of 2.38 Å for State 1, 2.42 Å for State 2 and 2.32 Å for NAC bound RNCs (Supplementary Fig. 10c-g, Supplementary Table 2).

In all cases cryo-EM densities were visualized with ChimeraX (v1.8)^56^.

### Model building

For the short (10aa) ARF1-linker RNC with NMT1 and NAC, the overall ribosome model from PDB: 6Y2L^57^ and proteins eS12 and eS31 from PDB: 8GLP^58^ were rigid body docked in the cryo-EM density. PDB: 5A8L^27^ was used as initial model for eRF1, P-site tRNA and the hCMV-stalling peptide. The hCMV peptide as wells as the other segments of our NMT1 substrate peptide were rebuild manually. While we were able to trace the nascent chain to NMT1 for some parts, like the connection between peptide tunnel exit and NMT1 the resolution did not allow for definitive amino acid placement. Therefore, we removed amino acid side chains at areas of limited resolution but left the nascent to illustrate its general path. The prolyl-tRNA decoding CTT was rebuild using HGNC:34631 as reference sequence without base modifications and mRNA was de novo built in Coot^59^. The P-stalk rRNA model was extracted from PDB: 8YOP^60^ and manually adjusted. For P-stalk proteins uL10 and uL11, models from the AF2^24^ database were docked and adjusted. The NAC complex model was taken from PDB: 7QWR^35^. The NAC globular domain was only rigidly docked due to the lower local resolution and the NACB anchor manually adjusted. A substrate bound crystal structure (PDB: 5O9U^6^) was used for NMT1 and the C-terminus of NACB was docked according to the AF2 multimer prediction^23,24^. Then, the combined model was real-space refined in Phenix^61^ (v 1.21.2-5419) (Supplementary Table 2) and manually adjusted in Coot^59^.

For the native NMT1 dataset, the combined translational states mostly resemble a hibernating ribosome with EEF2, E-site tRNA and SERBP1. Therefore, PDB: 6Z6M^18^ was used for 40S and 60S as well as hibernation factors and rigid body docked into the cryo-EM map. The model for NAC and NMT1 was taken from the higher resolved in vitro structure and also rigid body docked. Finally, the resulting model was manually adjusted in Coot^59^ and real-space refined in Phenix^61^ (Supplementary Table 2).

For the full length ARF1 RNCs with NMT1 and NAC in the canonical position (FL-State 1 and 2), the model for the short linker RNC (see above) was used as basis. NMT1 with substrate and NACB C-terminus was fitted separately and the NAC complex globular domain was substituted with an AF3 prediction^62^ and rigidly docked. For the ARF1 core fold PDB:8SDW^63^ was used. Due to the limited local resolution (Supplementary Fig. 8h, i) it was positioned with respect to density connecting the GTPase domain with the nascent chain and density protruding from the substrate binding cavity of NMT1 allowing for approximate placement of the respective C- and N-termini. However, the fitted ARF1 domain serves only as a representative model and could not be placed unambiguously. For the full length ARF1 RNC with alternative ARF1-NMT1-NAC conformation, also the short linker RNC model was used as starting point. Again, NMT1 with substrate and the NACB C-terminus was rigid body docked and the AF3 model of the NAC globular domain placed. Here, the orientation of ARF1 was more clearly distinguishable and was taken from the AF2 database as it allowed for a better fit compared to existing crystal structures. In all three cases models were manually adjusted in Coot^59^ and real-space refined in Phenix^61^ (Supplementary Table 2).

For the 58aa-ARF1 intermediate RNCs with NMT1 and NAC in 58aa-States 1 and 2, the model for the short ARF1 linker RNC (see above) was used as a starting point. NMT1 with NACB C-terminus and the substrate sequence, and the NAC globular domain were separately rigid body fitted. For the 58aa-ARF1 intermediate the AF3 prediction for residues 2-55aa of ARF1 were used and fitted into the extra density at the NAC β-sandwich. The remaining residues (56-59aa) were fitted separately for State 2 and omitted for State 1. Due to the low local resolution (Supplementary Fig. 10h, i) the intermediate substrate model docked only servers as placeholder likely representative for the density observed. For the NAC-bound 58aa-ARF1 RNC subclass, again the short linker RNC (see above) was used as initial model. The NAC globular domain was taken from PDB: 7QWR^35^ and rigid body docked. The nascent chain was rebuilt and extended as far as the density protruding from the peptide exit allowed. All models were then manually adjusted in Coot^59^ and real-space refined in Phenix^61^ (Supplementary Table 2).

In all cases molecular models were visualized with ChimeraX (v1.8)^56^.

### Statistics and reproducibility

Each representative biochemical experiment was repeated at least once with highly similar results. Cryo-EM data for each sample was only collected once. For in cellulo experiments, experiments were performed in triplicates. Mean values are displayed and error bars represent the standard error of the mean (SEM) as described in the figure legends.

### Reporting summary

Further information on research design is available in the Nature Portfolio Reporting Summary linked to this article.

## Supplementary information

Supplementary Information

Peer Review file

Reporting Summary

## Source data

Source Data

## Data Availability

Cryo-EM maps and molecular models generated in the study were deposited at the Electron Microscopy Data Bank (EMDB) or the Protein Data Bank (PDB), respectively, and are accessible via the following codes: EMD-52581 and 9I2D (NMT1-NAC bound human RNC with 10 amino acid ARF1-linker); EMD-52582 and 9I2E (NMT1-NAC bound human ribosome (combined translational states)); EMD-53230 and 9QLO (NMT1-NAC bound human RNC with full length ARF1 - State 1); EMD-53231 and 9QLP (NMT1-NAC bound human RNC with full length ARF1 - State 2); EMD-53232 and 9QLQ (NMT1-NAC bound human RNC with full length ARF1 - alternative State); EMD-54528 and 9S3B (NMT1-NAC bound human RNC with 58 amino acid ARF1-linker - State 1); EMD-54529 and 9S3C (NMT1-NAC bound human RNC with 58 amino acid ARF1-linker - State 2); EMD-54530 and 9S3D (NAC bound human RNC with 58 amino acid ARF1-linker). Source data are provided with this paper.

## References

[1] Gamerdinger, M. & Deuerling, E. Cotranslational sorting and processing of newly synthesized proteins in eukaryotes. *Trends Biochem Sci.* **49**, 105–118 (2024). 37919225 10.1016/j.tibs.2023.10.003

[2] Oye, H., Lundekvam, M., Caiella, A., Hellesvik, M. & Arnesen, T. Protein N-terminal modifications: molecular machineries and biological implications. *Trends Biochem Sci.* **50**, 290–310 (2025). 39837675 10.1016/j.tibs.2024.12.012

[3] Meinnel, T., Dian, C. & Giglione, C. Myristoylation, an ancient protein modification mirroring eukaryogenesis and evolution. *Trends Biochem Sci.* **45**, 619–632 (2020). 32305250 10.1016/j.tibs.2020.03.007

[4] Giglione, C. & Meinnel, T. Mapping the myristoylome through a complete understanding of protein myristoylation biochemistry. *Prog. Lipid Res* **85**, 101139 (2022). 34793862 10.1016/j.plipres.2021.101139

[5] Timms, R. T. et al. A glycine-specific N-degron pathway mediates the quality control of protein N-myristoylation. *Science* **365**10.1126/science.aaw4912 (2019). 10.1126/science.aaw4912 PMC7090375 31273098

[6] Castrec, B. et al. Structural and genomic decoding of human and plant myristoylomes reveals a definitive recognition pattern. *Nat. Chem. Biol.* **14**, 671–679 (2018). 29892081 10.1038/s41589-018-0077-5

[7] Meinnel, T. Comment on “Binding Affinity Determines Substrate Specificity and Enables Discovery of Substrates for N-Myristoyltransferases. *ACS Catal.* **12**, 8195–8201 (2022). 10.1021/acscatal.2c01818 PMC9361279 35966602

[8] Wilcox, C., Hu, J. S. & Olson, E. N. Acylation of proteins with myristic acid occurs cotranslationally. *Science* **238**, 1275–1278 (1987). 3685978 10.1126/science.3685978

[9] Magee, A. I. & Courtneidge, S. A. Two classes of fatty acid acylated proteins exist in eukaryotic cells. *EMBO J.* **4**, 1137–1144 (1985). 2988939 10.1002/j.1460-2075.1985.tb03751.x PMC554315

[10] Olson, E. N. & Spizz, G. Fatty acylation of cellular proteins. Temporal and subcellular differences between palmitate and myristate acylation. *J. Biol. Chem.* **261**, 2458–2466 (1986). 3944142

[11] Giang, D. K. & Cravatt, B. F. A second mammalian N-myristoyltransferase. *J. Biol. Chem.* **273**, 6595–6598 (1998). 9506952 10.1074/jbc.273.12.6595

[12] Tate, E. W., Soday, L., de la Lastra, A. L., Wang, M. & Lin, H. Protein lipidation in cancer: mechanisms, dysregulation and emerging drug targets. *Nat. Rev. Cancer* **24**, 240–260 (2024). 38424304 10.1038/s41568-024-00666-x

[13] Sangha, R. et al. Novel, first-in-human, Oral PCLX-001 treatment in a patient with relapsed diffuse large B-cell lymphoma. *Curr. Oncol.* **29**, 1939–1946 (2022). 35323358 10.3390/curroncol29030158 PMC8947478

[14] Riviere, F. et al. Novel, tightly structurally related N-myristoyltransferase inhibitors display equally potent yet distinct inhibitory mechanisms. *Structure* **32**, 1737–1750.e1733 (2024). 39208793 10.1016/j.str.2024.08.001

[15] Dian, C. et al. High-resolution snapshots of human N-myristoyltransferase in action illuminate a mechanism promoting N-terminal Lys and Gly myristoylation. *Nat. Commun.* **11**, 1132 (2020). 32111831 10.1038/s41467-020-14847-3 PMC7048800

[16] Riviere, F. et al. Structural and large-scale analysis unveil the intertwined paths promoting NMT-catalyzed lysine and glycine myristoylation. *J. Mol. Biol.* **434**, 167843 (2022). 36181773 10.1016/j.jmb.2022.167843

[17] Wiedmann, B., Sakai, H., Davis, T. A. & Wiedmann, M. A protein complex required for signal-sequence-specific sorting and translocation. *Nature* **370**, 434–440 (1994). 8047162 10.1038/370434a0

[18] Wells, J. N. et al. Structure and function of yeast Lso2 and human CCDC124 bound to hibernating ribosomes. *PLoS Biol.* **18**, e3000780 (2020). 32687489 10.1371/journal.pbio.3000780 PMC7392345

[19] Knorr, A. G. et al. Ribosome-NatA architecture reveals that rRNA expansion segments coordinate N-terminal acetylation. *Nat. Struct. Mol. Biol.* **26**, 35–39 (2019). 30559462 10.1038/s41594-018-0165-y

[20] Knorr, A. G. et al. The dynamic architecture of Map1- and NatB-ribosome complexes coordinates the sequential modifications of nascent polypeptide chains. *PLoS Biol.* **21**, e3001995 (2023). 37079644 10.1371/journal.pbio.3001995 PMC10118133

[21] Lentzsch, A. M. et al. NAC guides a ribosomal multienzyme complex for nascent protein processing. *Nature* **633**, 718–724 (2024). 39169182 10.1038/s41586-024-07846-7 PMC12039536

[22] Klein, M., Wild, K. & Sinning, I. Multi-protein assemblies orchestrate co-translational enzymatic processing on the human ribosome. *Nat. Commun.* **15**, 7681 (2024). 39227397 10.1038/s41467-024-51964-9 PMC11372111

[23] Evans, R. et al. Protein complex prediction with AlphaFold-Multimer. *bioRxiv*, 2021.2010.2004.463034 10.1101/2021.10.04.463034 (2022).

[24] Jumper, J. et al. Highly accurate protein structure prediction with AlphaFold. *Nature* **596**, 583–589 (2021). 34265844 10.1038/s41586-021-03819-2 PMC8371605

[25] Gamerdinger, M. et al. NAC controls cotranslational N-terminal methionine excision in eukaryotes. *Science* **380**, 1238–1243 (2023). 37347872 10.1126/science.adg3297

[26] Glover, C. J., Hartman, K. D. & Felsted, R. L. Human N-myristoyltransferase amino-terminal domain involved in targeting the enzyme to the ribosomal subcellular fraction. *J. Biol. Chem.* **272**, 28680–28689 (1997). 9353336 10.1074/jbc.272.45.28680

[27] Matheisl, S., Berninghausen, O., Becker, T. & Beckmann, R. Structure of a human translation termination complex. *Nucleic Acids Res.* **43**, 8615–8626 (2015). 26384426 10.1093/nar/gkv909 PMC4605324

[28] Deichaite, I., Casson, L. P., Ling, H. P. & Resh, M. D. In vitro synthesis of pp60v-src: myristylation in a cell-free system. *Mol. Cell Biol.* **8**, 4295–4301 (1988). 3141787 10.1128/mcb.8.10.4295-4301.1988 PMC365502

[29] Klein, M. A., Wild, K., Kisonaite, M. & Sinning, I. Methionine aminopeptidase 2 and its autoproteolysis product have different binding sites on the ribosome. *Nat. Commun.* **15**, 716 (2024). 38267453 10.1038/s41467-024-44862-7 PMC10808355

[30] Frottin, F. et al. MetAP1 and MetAP2 drive cell selectivity for a potent anti-cancer agent in synergy, by controlling glutathione redox state. *Oncotarget* **7**, 63306–63323 (2016). 27542228 10.18632/oncotarget.11216 PMC5325365

[31] Ross, S., Giglione, C., Pierre, M., Espagne, C. & Meinnel, T. Functional and developmental impact of cytosolic protein N-terminal methionine excision in Arabidopsis. *Plant Physiol.* **137**, 623–637 (2005). 15681659 10.1104/pp.104.056861 PMC1065363

[32] Pierre, M. et al. N-myristoylation regulates the SnRK1 pathway in Arabidopsis. *Plant Cell* **19**, 2804–2821 (2007). 17827350 10.1105/tpc.107.051870 PMC2048702

[33] Li, X. & Chang, Y. H. Amino-terminal protein processing in Saccharomyces cerevisiae is an essential function that requires two distinct methionine aminopeptidases. *Proc. Natl. Acad. Sci. USA* **92**, 12357–12361 (1995). 8618900 10.1073/pnas.92.26.12357 PMC40356

[34] Goldberg, J. Structural basis for activation of ARF GTPase: Mechanisms of guanine nucleotide exchange and GTP-myristoyl switching. *Cell* **95**, 237–248 (1998). 9790530 10.1016/s0092-8674(00)81754-7

[35] Jomaa, A. et al. Mechanism of signal sequence handover from NAC to SRP on ribosomes during ER-protein targeting. *Science* **375**, 839–844 (2022). 35201867 10.1126/science.abl6459 PMC7612438

[36] Noriega, T. R. et al. Signal recognition particle-ribosome binding is sensitive to nascent chain length. *J. Biol. Chem.* **289**, 19294–19305 (2014). 24808175 10.1074/jbc.M114.563239 PMC4094042

[37] Schibich, D. et al. Global profiling of SRP interaction with nascent polypeptides. *Nature* **536**, 219–223 (2016). 27487212 10.1038/nature19070

[38] Chartron, J. W., Hunt, K. C. & Frydman, J. Cotranslational signal-independent SRP preloading during membrane targeting. *Nature* **536**, 224–228 (2016). 27487213 10.1038/nature19309 PMC5120976

[39] Rabl, L. & Deuerling, E. The nascent polypeptide-associated complex (NAC) as regulatory hub on ribosomes. *Biol. Chem*. 10.1515/hsz-2025-0114 (2025). 10.1515/hsz-2025-0114 40167342

[40] Shen, K. et al. Dual role of ribosome-binding domain of NAC as a potent suppressor of protein aggregation and aging-related proteinopathies. *Mol. Cell* **74**, 729–741.e727 (2019). 30982745 10.1016/j.molcel.2019.03.012 PMC6527867

[41] Koplin, A. et al. A dual function for chaperones SSB-RAC and the NAC nascent polypeptide-associated complex on ribosomes. *J. Cell Biol.* **189**, 57–68 (2010). 20368618 10.1083/jcb.200910074 PMC2854369

[42] Kirstein-Miles, J., Scior, A., Deuerling, E. & Morimoto, R. I. The nascent polypeptide-associated complex is a key regulator of proteostasis. *EMBO J.* **32**, 1451–1468 (2013). 23604074 10.1038/emboj.2013.87 PMC3655472

[43] Santos, J. et al. NAC promotes co-translational protein folding at the ribosomal tunnel exit. *bioRxiv*10.1101/2025.08.02.668148 (2025). 10.1016/j.molcel.2026.02.022 PMC13020645 41875886

[44] Gamerdinger, M. et al. Mechanism of cotranslational protein N-myristoylation in human cells. *Mol. Cell* **85**, 2749–2758.e2748 (2025). 40639378 10.1016/j.molcel.2025.06.015 PMC12863714

[45] Zdancewicz, S., Maldosevic, E., Malezyna, K. & Jomaa, A. NAC couples protein synthesis with nascent polypeptide myristoylation on the ribosome. *EMBO J*. 10.1038/s44318-025-00548-4 (2025). 10.1038/s44318-025-00548-4 PMC12623983 40859030

[46] Su, D., Kosciuk, T., Yang, M., Price, I. R. & Lin, H. Binding affinity determines substrate specificity and enables discovery of substrates for N-myristoyltransferases. *ACS Catal.* **11**, 14877–14883 (2021). 34956690 10.1021/acscatal.1c03330 PMC8689648

[47] Papasergi-Scott, M. M. et al. Structures of Ric-8B in complex with Galpha protein folding clients reveal isoform specificity mechanisms. *Structure* **31**, 553–564.e557 (2023). 36931277 10.1016/j.str.2023.02.011 PMC10164081

[48] Seven, A. B. et al. Structures of galpha proteins in complex with their chaperone reveal quality control mechanisms. *Cell Rep.* **30**, 3699–3709.e3696 (2020). 32126208 10.1016/j.celrep.2020.02.086 PMC7192526

[49] Monassa, P. et al. Biochemical and structural analysis of N-myristoyltransferase mediated protein tagging. *Methods Enzymol.* **684**, 135–166 (2023). 37230587 10.1016/bs.mie.2023.02.016

[50] Narita, M. et al. A distinct mammalian disome collision interface harbors K63-linked polyubiquitination of uS10 to trigger hRQT-mediated subunit dissociation. *Nat. Commun.* **13**, 6411 (2022). 36302773 10.1038/s41467-022-34097-9 PMC9613687

[51] Zheng, S. Q. et al. MotionCor2: anisotropic correction of beam-induced motion for improved cryo-electron microscopy. *Nat. Methods* **14**, 331–332 (2017). 28250466 10.1038/nmeth.4193 PMC5494038

[52] Rohou, A. & Grigorieff, N. CTFFIND4: Fast and accurate defocus estimation from electron micrographs. *J. Struct. Biol.* **192**, 216–221 (2015). 26278980 10.1016/j.jsb.2015.08.008 PMC6760662

[53] Wagner, T. et al. SPHIRE-crYOLO is a fast and accurate fully automated particle picker for cryo-EM. *Commun. Biol.* **2**, 218 (2019). 31240256 10.1038/s42003-019-0437-z PMC6584505

[54] Kimanius, D., Dong, L., Sharov, G., Nakane, T. & Scheres, S. H. W. New tools for automated cryo-EM single-particle analysis in RELION-4.0. *Biochem J.* **478**, 4169–4185 (2021). 34783343 10.1042/BCJ20210708 PMC8786306

[55] Punjani, A., Rubinstein, J. L., Fleet, D. J. & Brubaker, M. A. cryoSPARC: algorithms for rapid unsupervised cryo-EM structure determination. *Nat. Methods* **14**, 290–296 (2017). 28165473 10.1038/nmeth.4169

[56] Meng, E. C. et al. UCSF ChimeraX: Tools for structure building and analysis. *Protein Sci.* **32**, e4792 (2023). 37774136 10.1002/pro.4792 PMC10588335

[57] Bhaskar, V. et al. Dynamics of uS19 C-terminal tail during the translation elongation cycle in human ribosomes. *Cell Rep.* **31**, 107473 (2020). 32268098 10.1016/j.celrep.2020.03.037

[58] Holm, M. et al. mRNA decoding in human is kinetically and structurally distinct from bacteria. *Nature* **617**, 200–207 (2023). 37020024 10.1038/s41586-023-05908-w PMC10156603

[59] Emsley, P. & Cowtan, K. Coot: Model-building tools for molecular graphics. *Acta Crystallogr D. Biol. Crystallogr* **60**, 2126–2132 (2004). 15572765 10.1107/S0907444904019158

[60] Li, X. et al. Structural basis for differential inhibition of eukaryotic ribosomes by tigecycline. *Nat. Commun.* **15**, 5481 (2024). 38942792 10.1038/s41467-024-49797-7 PMC11213857

[61] Liebschner, D. et al. Macromolecular structure determination using X-rays, neutrons and electrons: recent developments in Phenix. *Acta Crystallogr D. Struct. Biol.* **75**, 861–877 (2019). 31588918 10.1107/S2059798319011471 PMC6778852

[62] Abramson, J. et al. Accurate structure prediction of biomolecular interactions with AlphaFold 3. *Nature* **630**, 493–500 (2024). 38718835 10.1038/s41586-024-07487-w PMC11168924

[63] Rosenberg, E. M. Jr et al. Point mutations in Arf1 reveal cooperative effects of the N-terminal extension and myristate for GTPase-activating protein catalytic activity. *PLoS One* **19**, e0295103 (2024). 38574162 10.1371/journal.pone.0295103 PMC10994351

