# Supplementary material for: Structural basis of co-translational N-myristoylation in humans
Source: Nat Commun. 2026 Jan 23;17:1191. doi: 10.1038/s41467-025-67962-4 (PMC12858966; doi:10.1038/s41467-025-67962-4)
Supplement: Supplementary file 1 — Supplementary Information [file 41467_2025_67962_MOESM1_ESM.pdf]

Supplementary Information

Structural basis of co-translational N-myristoylation in humans

Supplementary Table 1: In cellulo stoichiometries of the main protein partners involved in myristoylation at the ribosome

Protein or complex	Concentration (nM)*	Stoichiometry£
NMT1	186	0.5
NMT2	2	0.0
METAP1	203	0.5
METAP2	346	0.8
NatA§	374	0.9
NAC§	408	1.0
Ribosome$	2506	6.1
Nascent chains!	1754	4.3
MYR nascent chains (NMT substrates)&	35	0.1
NatA-nascent chain substrates@	702	1.7
ARF1	1051	2.6

* data from HeLa cells (see Table S3 in [1]).

§ whenever a protein complex is considered, the concentration of the limiting subunit - that is, the one present at the lowest concentration required for ribosome binding - is considered (here BTF3/NACB for NAC and NAA15 for NatA)

$ average concentration of 51 reported 60S protein components

£ a value of 1 was given to NAC

! considering 70% active ribosomes

& considering 2% myristoylated proteins and that their average abundance reflects that of the entire proteome (validated with 211 matching NMT substrates over 8803 proteins quantified)

@ considering 40% proteins as NatA substrates

Supplementary Table 2: Data collection details and model refinement statistics

	NMT1-NAC bound human RNC with 10 amino acid ARF1-linker<br>PDB: 9I2D<br>EMD-52581	NMT1-NAC bound human ribosome (combined translational states)<br>PDB: 9I2E<br>EMD-52582
**Data collection and processing**		
Micrographs (no.)	38,099	13,761
Magnification	165,000	130,000
Voltage (kV)	300	300
Electron exposure ($e^-/Å^2$)	40	45.6
Defocus range (μm)	0.5-3.5	0.5-3.5
Pixel size (Å)	0.727	1.049
Symmetry imposed	C1	C1
Initial particle images (no.)	788,761	215,195
Final particle images (no.)	163,265	60,808
Map resolution (Å)	2.19	2.95
FSC threshold	0.143	0.143
Masked local resolution range (Å)	1.6-34.9	2.3-44.5
Map sharpening *B* factor ($Å^2$)	-42.7	-71.6
**Refinement**		
Initial models used (PDB code)	6Y2L,8GLP, 5A8L, 8YOP, 7QWR,5O9U, AlphaFold 2	6Z6M, 10aa linker in vitro model
Model resolution (Å)	2.2	2.9
FSC threshold	0.5	0.5
Model composition		
Non-hydrogen atoms	222,257	230,823
Protein residues	12,719	13,179
Nucleotide residues	5,724	5,873
R.m.s. deviations		
Bond lengths (Å)	0.006	0.005
Bond angles (°)	0.930	0.953
Validation		
MolProbity score	1.80	2.25
Clashscore	2.93	5.39
Poor rotamers (%)	3.05	4.68
Ramachandran plot		
Favored (%)	94.88	92.92
Allowed (%)	5.03	7.05
Disallowed (%)	0.10	0.03
Map vs. Model CC	0.91	0.85

Supplementary Table 2: Data collection details and model refinement statistics; continued

	NMT1-NAC bound human RNC with full length ARF1 - State 1<br><br>PDB: 9QLO<br>EMD-53230	NMT1-NAC bound human RNC with full length ARF1 - State 2<br><br>PDB: 9QLP<br>EMD-53231	NMT1-NAC bound human RNC with full length ARF1 – alternative State<br>PDB: 9QLQ<br>EMD-53232
**Data collection and processing**			
Micrographs (no.)	57,603		
Magnification	165,000		
Voltage (kV)	300		
Electron exposure ($e^-/Å^2$)	40		
Defocus range (µm)	0.5-3.5		
Pixel size (Å)	0.727		
Symmetry imposed	C1		
Initial particle images (no.)	1,115,225		
Final particle images (no.)	62,249	21,642	44,109
Map resolution (Å)	2.47	2.75	2.57
FSC threshold	0.143	0.143	0.143
Masked local resolution range (Å)	1.6-38.7	1.6-42.6	1.6-42.6
Map sharpening *B* factor ($Å^2$)	-54.3	-47.8	-53.0
**Refinement**			
Initial models used (PDB code)	10aa linker in vitro model, 8SDW, AF3	10aa linker in vitro model, 8SDW, AF3	10aa linker in vitro model, AF2, AF3
Model resolution (Å)	2.5	2.8	2.6
FSC threshold	0.5	0.5	0.5
Model composition			
Non-hydrogen atoms	223,462	223,419	223,423
Protein residues	12,872	13,179	12,864
Nucleotide residues	5,724	5,724	5,724
R.m.s. deviations			
Bond lengths (Å)	0.004	0.004	0.06
Bond angles (°)	0.841	0.823	0.838
Validation			
MolProbity score	1.80	1.72	1.54
Clashscore	3.15	3.10	3.85
Poor rotamers (%)	2.82	2.27	0.00
Ramachandran plot			
Favored (%)	94.88	94.93	94.70
Allowed (%)	5.07	5.03	5.22
Disallowed (%)	0.05	0.03	0.08
Map vs. Model CC	0.89	0.88	0.86

Supplementary Table 2: Data collection details and model refinement statistics; continued

	NMT1-NAC bound human RNC with 58 amino acid ARF1-linker - State 1<br><br>PDB: 9S3B<br>EMD-54528	NMT1-NAC bound human RNC with 58 amino acid ARF1-linker - State 2<br><br>PDB: 9S3C<br>EMD-54529	NAC bound human RNC with 58 amino acid ARF1-linker<br><br>PDB: 9S3D<br>EMD-54530
**Data collection and processing**			
Micrographs (no.)	46,292		
Magnification	165,000		
Voltage (kV)	300		
Electron exposure (e$^-$/Å^2)	40		
Defocus range (µm)	0.5-3.5		
Pixel size (Å)	0.727		
Symmetry imposed	C1		
Initial particle images (no.)	1,008,730		
Final particle images (no.)	60,443	49,105	74,754
Map resolution (Å)	2.38	2.42	2.32
FSC threshold	0.143	0.143	0.143
Masked local resolution range (Å)	1.6-38.8	1.6-38.8	1.6-38.8
Map sharpening *B* factor (Å^2)	-35.6	-37.8	-52.5
**Refinement**			
Initial models used (PDB code)	10aa linker in vitro model, 8SDW, AF3	10aa linker in vitro model, 8SDW, AF3	10aa linker in vitro model, 7QWR
Model resolution (Å)	2.4	2.5	2.4
FSC threshold	0.5	0.5	0.5
Model composition			
Non-hydrogen atoms	222,378	222,376	218,816
Protein residues	12,744	12,745	12,291
Nucleotide residues	5,724	5,724	5,724
R.m.s. deviations			
Bond lengths (Å)	0.007	0.006	0.006
Bond angles (°)	0.923	0.870	0.886
Validation			
MolProbity score	1.92	1.93	1.90
Clashscore	4.06	4.27	4.81
Poor rotamers (%)	3.06	2.98	2.42
Ramachandran plot			
Favored (%)	94.69	94.74	94.77
Allowed (%)	5.22	5.20	5.19
Disallowed (%)	0.09	0.06	0.04
Map vs. Model CC	0.91	0.90	0.93

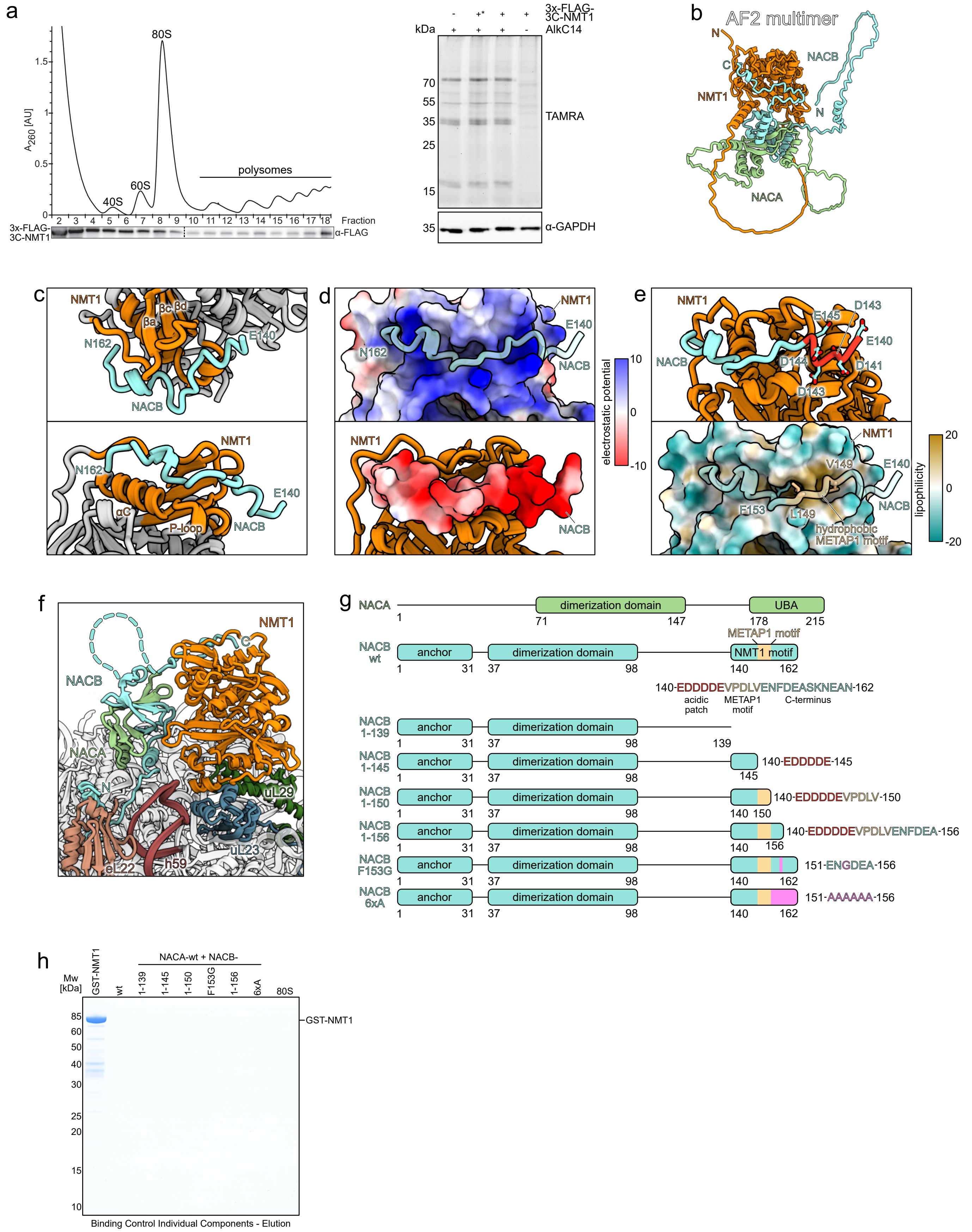

Supplementary Fig. 1: NMT1 in vivo polysome association, details of NMT1-NAC interaction prediction and in vitro binding assay. a, Polysome gradient profile from Expi293 cell lysate overexpressing 3xFLAG-3C-NMT1 (left). Western blot of gradient fractions with an antibody against the FLAG-tag (underneath). Click-chemistry analysis of influence of NMT1 overexpression on the myristoylated proteome (right). HEK293 cell lysates were prepared in the presence of alkyne-myristate (AlkC14), conjugated with TAMRA via click chemistry, separated by SDS-PAGE, and imaged by fluorescence. In control conditions, AlkC14 was substituted with myristate. The star indicates that an N-terminally truncated NMT1 variant (Δ1–115) was used. **b,** AF2 multimer prediction for NMT1, NACA and NACB isoform 1 (differs from isoform 2 by a 44 amino acid extended N-terminus). The termini of NACA as well as the globular domain are not predicted to participate in NMT1 binding, only the C-terminus of NACB. **c-e,** Details of the NMT1-NACB C-terminus interaction prediction. NACB residues are numbered according to isoform 2 used for in

vitro binding assays and in vitro reconstitution for cryo-EM. **c,** Detailed view of the NACB C-terminus-NMT1 interaction site. NMT1 elements forming the cavity for NACB C-terminus binding are shown. **d,** Surface representations of NMT1 (top) or the NACB C-terminus (bottom) colored by electrostatic potential. **e,** Focused view on the acidic patch (140-145aa) of the NACB C-terminus with the Cα backbone colored in red (top) and surface representation of NMT1 colored by lipophilicity with the hydrophobic METAP1 interaction motif (beige) and F153 inserted into a hydrophobic pocket of NMT1 (bottom). **f,** Overview of the NMT1-NAC assembly at the peptide tunnel exit of native ribosome complexes with relevant rRNA elements and ribosomal proteins highlighted. **g,** Schematic overview of the different NAC complexes used for in vitro binding assays and the domain organization of NACA and NACB is shown. C-terminal amino acid sequences are shown on the right for wildtype NACB as well as truncations and mutations. **h,** Coomassie-stained SDS-PAGE gel of the elution fractions (50%) of individual components used in the in vitro binding assay (see Fig. 1f). Source data are provided as a Source Data file.

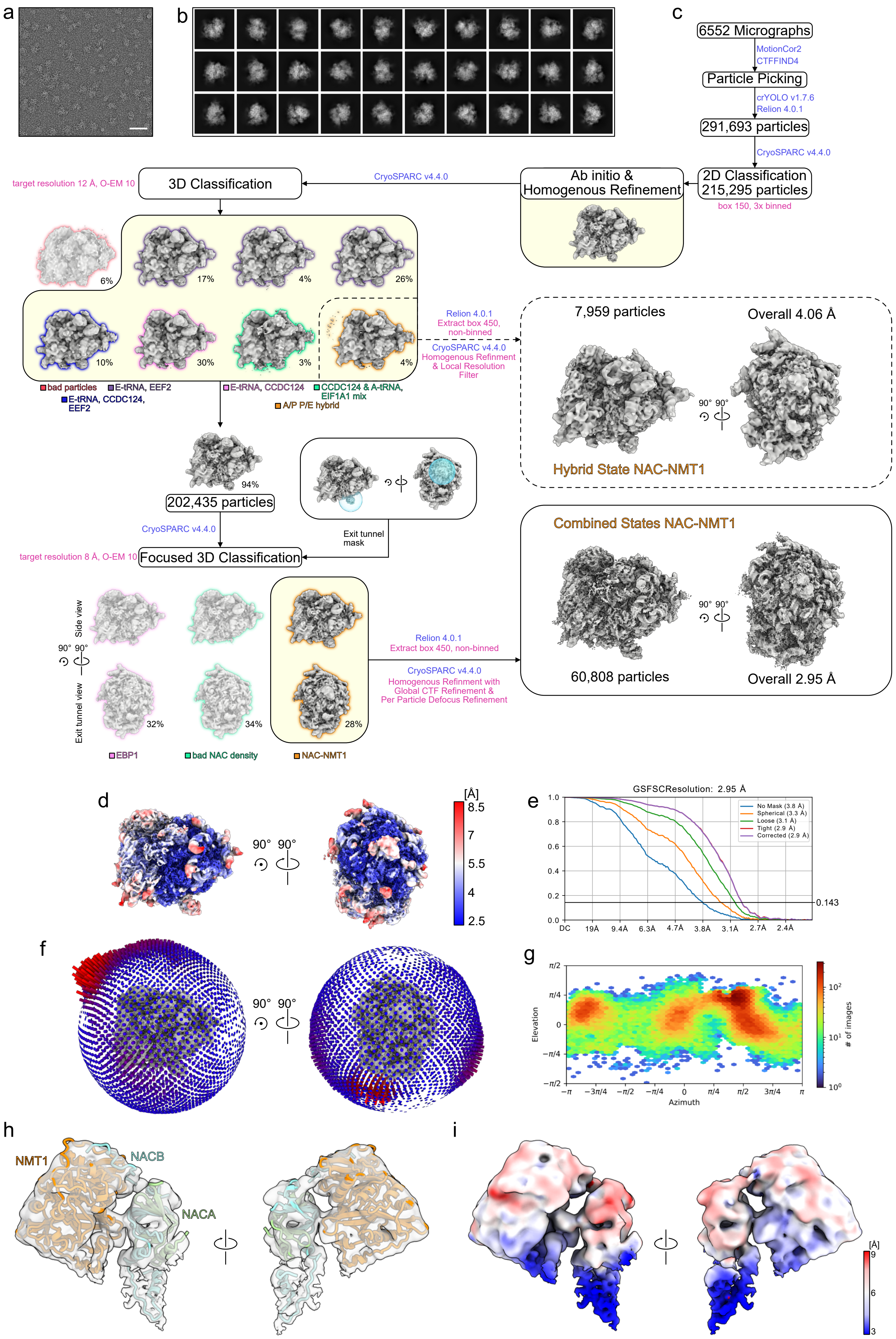

a
b
c
6552 Micrographs
MotionCor2
CTFFIND4
Particle Picking
crYOLO v1.7.6
Relion 4.0.1
291,693 particles
CryoSPARC v4.4.0
2D Classification
215,295 particles
box 150, 3x binned
Ab initio &
Homogenous Refinement
CryoSPARC v4.4.0
3D Classification
target resolution 12 Å, O-EM 10
6%
17%
4%
26%
10%
30%
3%
4%
bad particles
E-tRNA, EEF2
E-tRNA, CCDC124, EEF2
E-tRNA, CCDC124
CCDC124 & A-tRNA, EIF1A1 mix
A/P P/E hybrid
Relion 4.0.1
Extract box 450, non-binned
CryoSPARC v4.4.0
Homogenous Refinement & Local Resolution Filter
7,959 particles
Overall 4.06 Å
90° 90°
Hybrid State NAC-NMT1
94%
202,435 particles
CryoSPARC v4.4.0
Exit tunnel mask
target resolution 8 Å, O-EM 10
Focused 3D Classification
Side view
Exit tunnel view
32%
34%
28%
EBP1
bad NAC density
NAC-NMT1
Relion 4.0.1
Extract box 450, non-binned
CryoSPARC v4.4.0
Homogenous Refinment with Global CTF Refinement & Per Particle Defocus Refinement
Combined States NAC-NMT1
60,808 particles
Overall 2.95 Å
d
[Å]
8.5
7
5.5
4
2.5
e
GSFSCResolution: 2.95 Å
No Mask (3.8 Å)
Spherical (3.3 Å)
Loose (3.1 Å)
Tight (2.9 Å)
Corrected (2.9 Å)
0.143
DC 19Å 9.4Å 6.3Å 4.7Å 3.8Å 3.1Å 2.7Å 2.4Å
f
g
Elevation
Azimuth
# of images
h
NMT1
NACB
NACA
i
[Å]
9
6
3

Supplementary Fig. 2: Data processing for the native NMT1 pull-out cryo-EM dataset. a, Representative, filtered micrograph with 50 nm scale bar. **b,** Representative 2D class averages for RNCs. **c,** cryo-EM data processing and sorting scheme. Selected classes from 3D classifications are framed. Relevant processing steps are highlighted, parameters are noted in pink and software used in blue. **d,** cryo-EM map of the NMT1-bound combined translational states filtered and colored according to local resolution. **e,** gold standard Fourier shell correlation curves (GSFSC, as generated by cryoSPARC) with 0.143 criterion for overall resolution estimation. **f,** 3D representation for the orientation distribution of final particles. g, 2D heatmap for particle viewing direction and numbers as generated by cryoSPARC. **h,** Segmented, local resolution filtered density and molecular model for the NMT1-NAC exit-tunnel assembly. **i,** Segmented density shown in h colored according to local resolution.

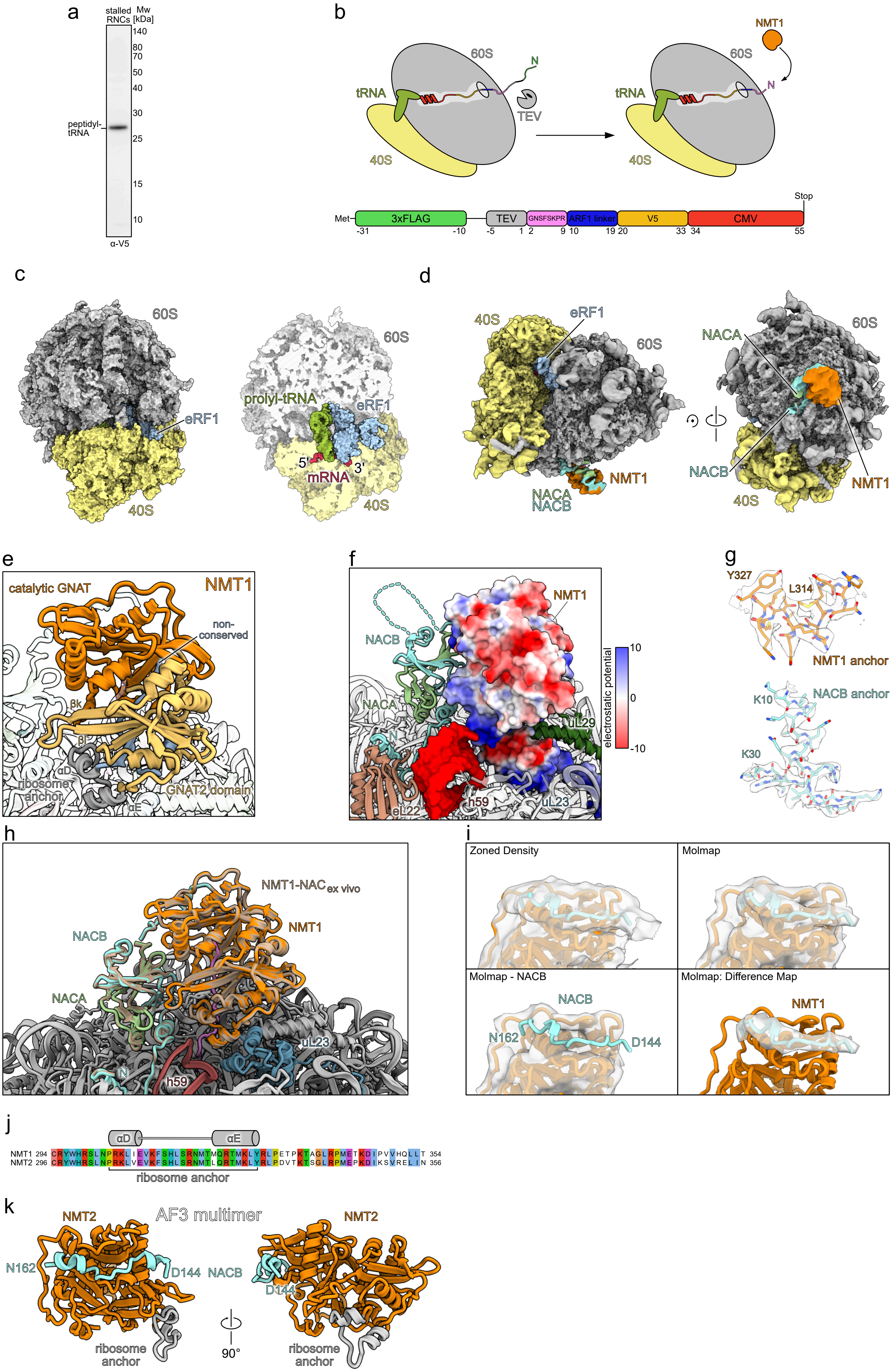
a
stalled RNCs
Mw [kDa]
140
80
70
50
40
30
25
15
10
peptidyl-tRNA
α-V5
b
NMT1
60S
N
tRNA
TEV
40S
Met
3xFLAG
TEV
GNSFSKPR
ARF1 linker
V5
CMV
Stop
-31
-10
-5
1
2
9
10
19
20
33
34
55
c
60S
eRF1
40S
prolyl-tRNA
5'
3'
mRNA
d
eRF1
NACA
NACB
NMT1
e
catalytic GNAT
NMT1
non-conserved
βk
βl
αD
αE
ribosome anchor
GNAT2 domain
f
NACB
NACA
N
uL29
eL22
h59
uL23
electrostatic potential
10
0
-10
g
Y327
L314
NMT1 anchor
NACB anchor
K10
K30
h
NMT1-NAC ex vivo
NMT1
NACB
NACA
uL23
N
h59
i
Zoned Density
Molmap
Molmap - NACB
Molmap: Difference Map
NACB
N162
D144
NMT1
j
αD
αE
NMT1 294 CRYWHRSLNPRKLIEVKFSHLSRNMTMQRTMKLYRLPETPKTAGLRPMETKDIPVVHQLLT 354
NMT2 296 CRYWHRSLNPRKLVEVKFSHLSRNMTLQRTMKLYRLPDVTKTSGLRPMEPKDIKSVRELIN 356
ribosome anchor
k
NMT2
AF3 multimer
N162
D144
NACB
ribosome anchor
90°

Supplementary Fig. 3: Details of NMT1-NAC bound RNCs. a, Western blot of purified RNCs with the short ARF1 (9-18aa) linker. The nascent polypeptide was detected via the encoded V5 peptide. The shift of the peptide product (5.9 kDa) to ~27 kDa indicates intact peptidyl-tRNA and stalling. The contrast was adjusted. **b,** Cartoon representation of a stalled ribosome with nascent polypeptide chain where TEV protease reveals the N-terminal NMT1 substrate sequence (top). Schematic representation of the nascent chain encoded by hCMV staller mRNA used to purify RNCs for cryo-EM (bottom). Amino acid numbering is annotated. The segments of the nascent chain and the NMT1 substrate sequence is shown. **c,** Surface representation of hCMV stalled RNCs (left) and cut-through view showing mRNA, tRNA and eRF1 in the intersubunit space (right). The presence of prolyl-tRNA and eRF1 indicate the correct stalling position. **d,** Colored, local resolution filtered cryo-EM density and molecular model of NMT1-NAC bound RNCs. **e,** Model of ribosome bound NMT1 with domains colored and the NMT1 ribosome anchor highlighted in grey. **f,** Molecular models of the NMT1 (top) and NACB (bottom) ribosome anchors and their corresponding isolated cryo-EM densities. **g,** Focused view of ribosome bound NMT1-NAC. Surface representations of NMT1, 28S rRNA h59 and uL23 were colored by electrostatic potential. **h,** Overlay of molecular models of the NMT1-NAC RNC and NMT1-NAC isolated from cells (brown). **i,** Comparison of experimental with calculated density for the NMT1-NACB C-terminus interaction. Density around NMT1-NACB C-terminus (top left) was compared with molmaps based on molecular models at 8 Å of NMT1 with (top right) or without NACB C-terminus (bottom left). A difference map between the two molmaps was generated (bottom right). The experimental density is closest to the theoretical density of NMT1 with the NACB C-terminus bound. **j,** Partial sequence alignment of human NMT1 and NMT2. The ribosome anchor motif with the alpha helices D and E are shown. **k,** AF3 multimer prediction of NMT1 with NAC. For clarity only the NAC the NACB C-terminus is shown. The C-terminus is numbered according to NACB isoform 2. Source data are provided as a Source Data file.

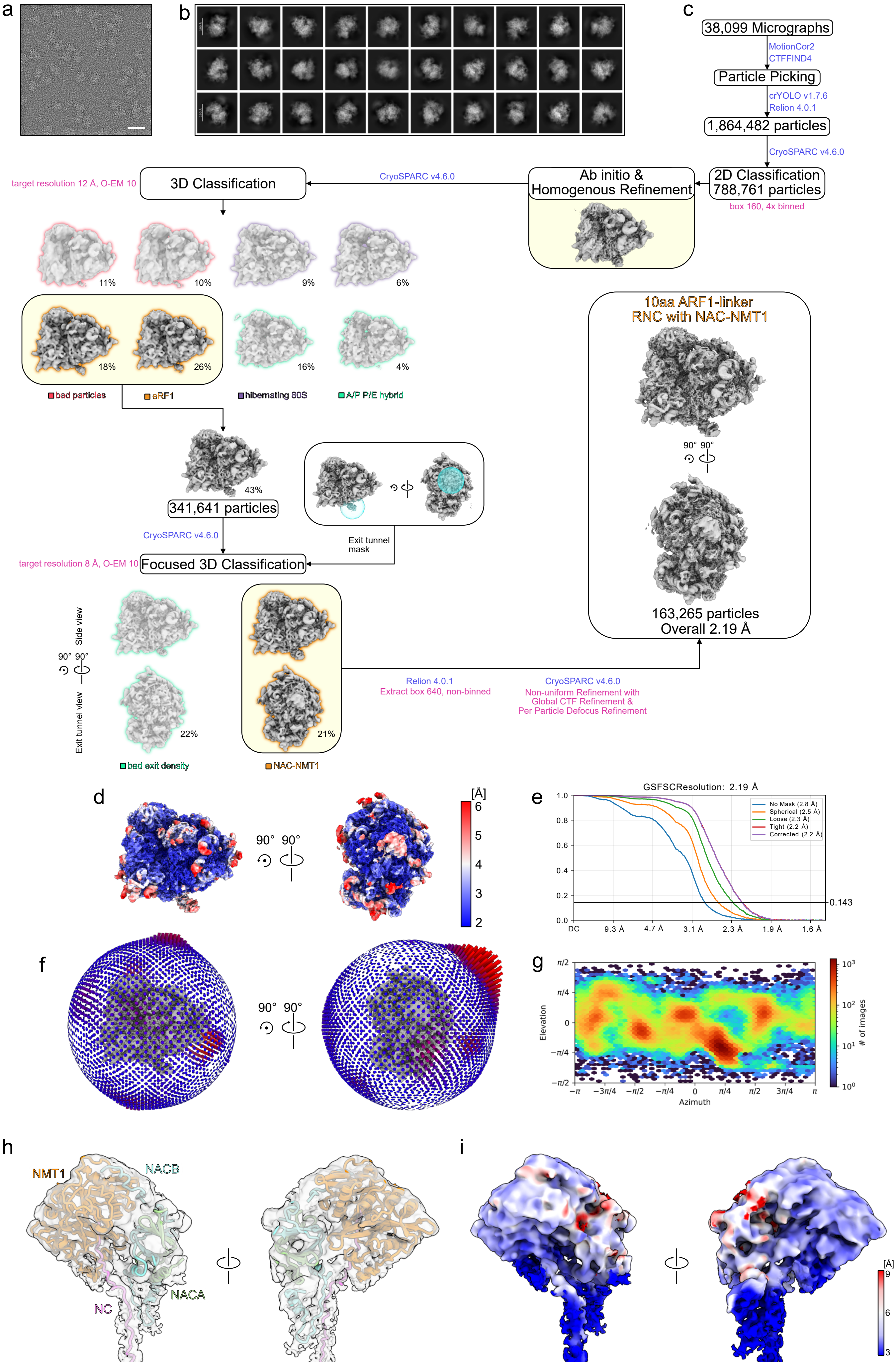

a
b
c
38,099 Micrographs
MotionCor2
CTFFIND4
Particle Picking
crYOLO v1.7.6
Relion 4.0.1
1,864,482 particles
CryoSPARC v4.6.0
2D Classification
788,761 particles
box 160, 4x binned
Ab initio &
Homogenous Refinement
CryoSPARC v4.6.0
3D Classification
target resolution 12 Å, O-EM 10
11%
10%
9%
6%
18%
26%
16%
4%
bad particles
eRF1
hibernating 80S
A/P P/E hybrid
43%
341,641 particles
CryoSPARC v4.6.0
Exit tunnel
mask
target resolution 8 Å, O-EM 10
Focused 3D Classification
Side view
90° 90°
Exit tunnel view
22%
21%
bad exit density
NAC-NMT1
Relion 4.0.1
Extract box 640, non-binned
CryoSPARC v4.6.0
Non-uniform Refinement with
Global CTF Refinement &
Per Particle Defocus Refinement
10aa ARF1-linker
RNC with NAC-NMT1
90° 90°
163,265 particles
Overall 2.19 Å
d
90° 90°
[Å]
6
5
4
3
2
e
GSFSCResolution: 2.19 Å
No Mask (2.8 Å)
Spherical (2.5 Å)
Loose (2.3 Å)
Tight (2.2 Å)
Corrected (2.2 Å)
0.143
DC
9.3 Å
4.7 Å
3.1 Å
2.3 Å
1.9 Å
1.6 Å
f
90° 90°
g
Elevation
Azimuth
# of images
h
NMT1
NACB
NACA
NC
i
[Å]
9
6
3

Supplementary Fig. 4: Data processing for the short ARF1 linker RNC-NMT1-NAC in vitro cryo-EM dataset. a, Representative, filtered micrograph with 35 nm scale bar. **b,** Representative 2D class averages for RNCs. **c,** cryo-EM data processing and sorting scheme. Selected classes from 3D classifications are framed. Relevant processing steps are highlighted, parameters are noted in pink and software used in blue. **d,** cryo-EM map of the NMT1-NAC-RNC filtered and colored according to local resolution. **e,** gold standard Fourier shell correlation curves (GSFSC, as generated by cryoSPARC) with 0.143 criterion for overall resolution estimation. **f,** 3D representation for the orientation distribution of final particles. **g,** 2D heatmap for particle viewing direction and numbers as generated by cryoSPARC. **h,** Segmented, local resolution filtered density and molecular model for the NMT1-NAC-nascent polypeptide exit-tunnel assembly. **i,** Segmented density shown in **h** colored according to local resolution.

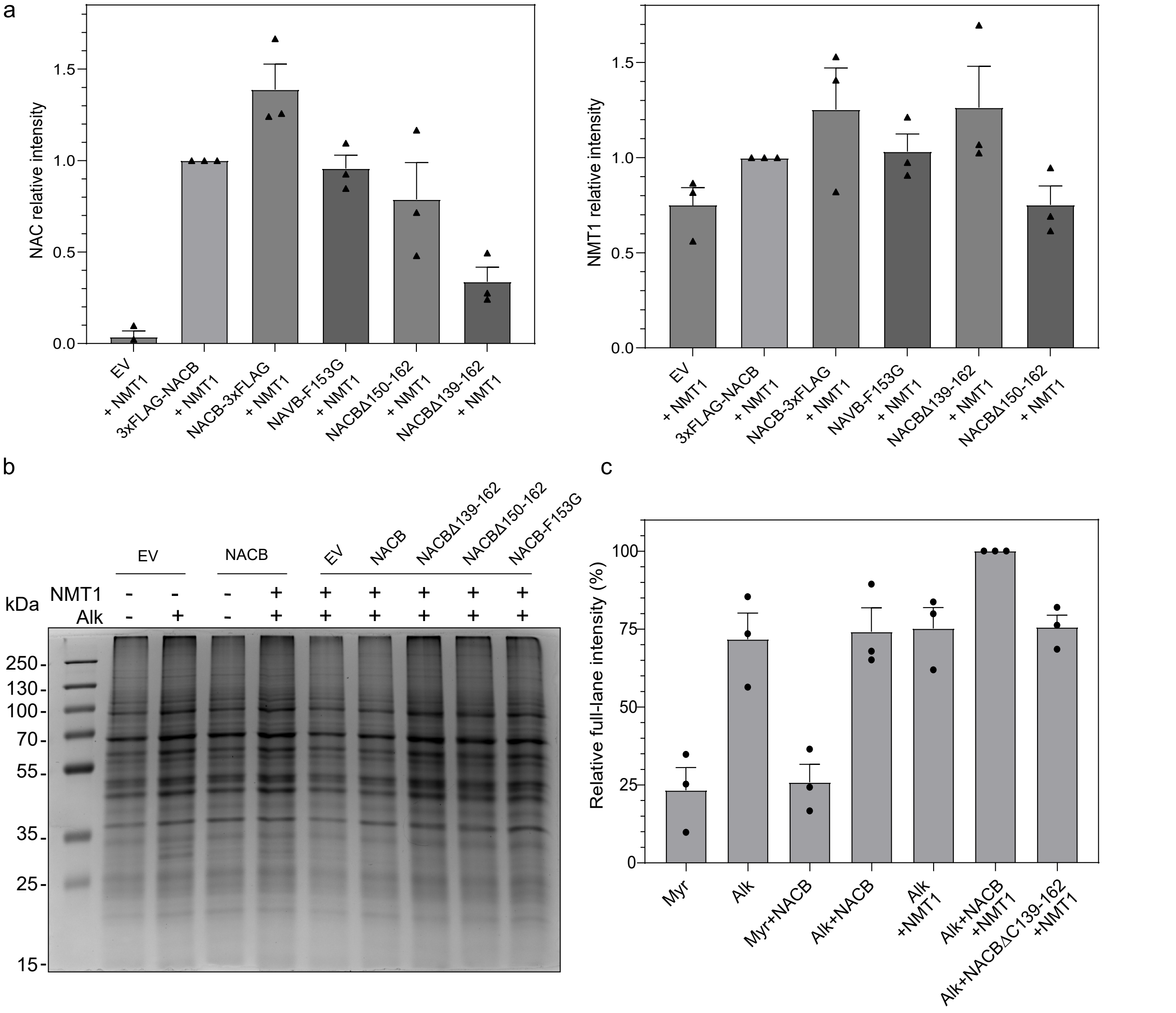

Supplementary Fig. 5: Functional investigation of NMT1-NAC RNC binding. n=3, mean values are displayed and error bars represent the standard error of the mean (SEM). **a,** quantification of Fig. 3c; average of three replicates. **b,** Coomassie blue staining of the denaturing SDS-PAGE gel shown in Fig. 3d. The first lane corresponds to the molecular weight marker. The size in kDa of each band is indicated on the left part. **c,** quantification of TAMRA fluorescence, all lanes, average of three replicates. The negative control lane (Myr) exhibits an average background of ~23% relative to the positive control (Alk + NACB), primarily due to several unspecific bands that account for ~18% of the signal (Fig. 3d). Source data are provided as a Source Data file.

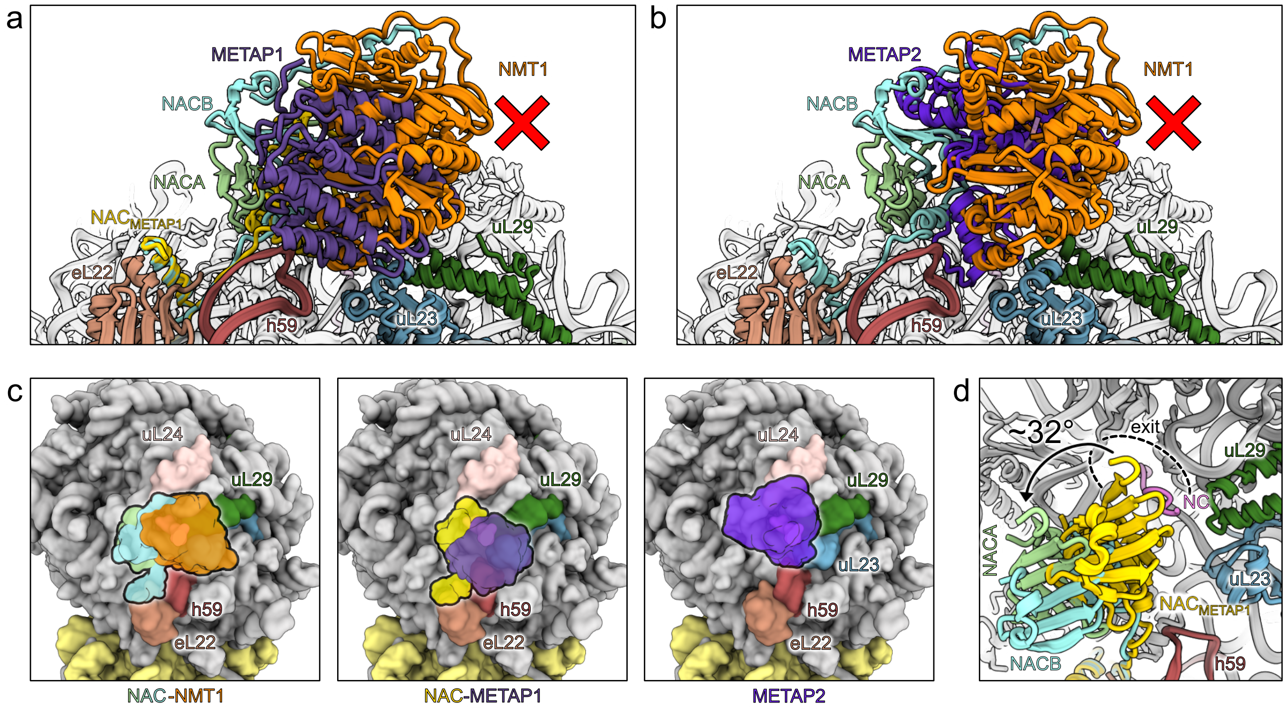

Supplementary Fig. 6: Mutually exclusive NMT1 and MetAP ribosome binding. a and b, Overlays of molecular models of ribosome bound NAC-METAP1 (PDB: 8P2K) (**a**) and METAP2 (PDB: 8ONY) (**b**) with ribosome bound NMT1-NAC (see Fig. 3c and d). Red crosses indicate overall clashes that prevent joint binding of MetAP and NMT1. **c,** Top views onto the ribosome exit tunnel site. Colored shapes of either NAC-NMT1, NAC-METAP1 or METAP2 are overlaid showing the area occupied by these factors, respectively. **d,** Comparison between NAC complex globular domain positioning for METAP1 (PDB: 8P2K) and NMT1. Overlaid molecular models are shown and NMT1 and METAP1 are excluded for clarity. The rotation for the globular domain from the METAP1 to the NMT1 state is indicated.

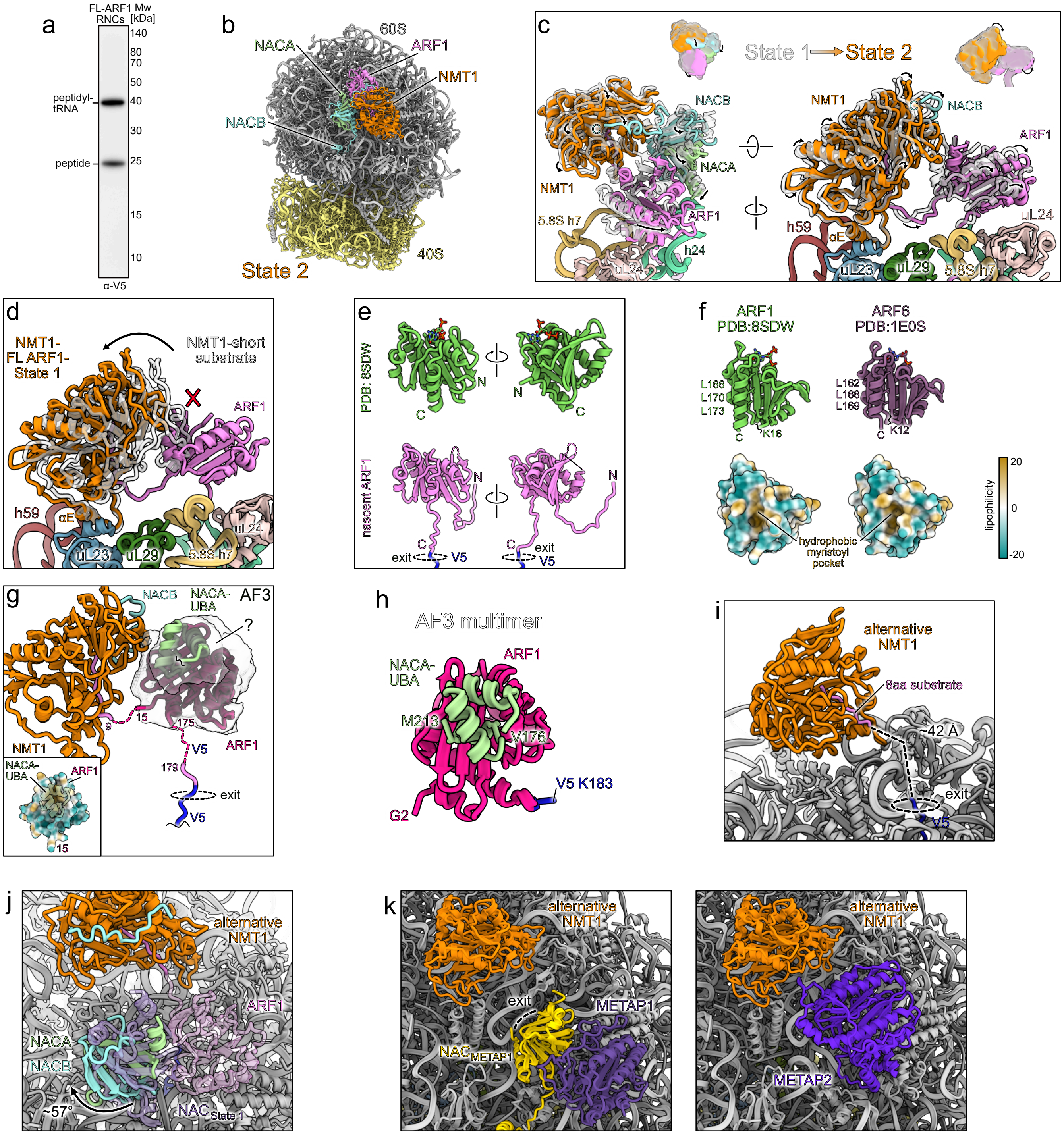

Supplementary Fig. 7: Details of reconstituted NMT1-NAC full-length ARF1 RNCs. a, Western blot of purified full-length ARF1 (9-181aa) linker RNCs. The nascent polypeptide was detected via the encoded V5 peptide. The shift of the peptide (24.5 kDa) to ~40 kDa indicates intact peptidyl-tRNA and ribosome stalling. The contrast was adjusted. **b,** Molecular model of full length ARF1 RNCs canonical NMT1 - State 2. **c,** Comparison of State 1 and 2 with State 1 overlaid in gray and transparent. Arrows indicate movement of factors from State 1 to 2. Cartoon representations in the top summarize the overall movements. **d,** Comparison between the conformation of NMT1 in the full length and short ARF1 (gray and transparent) RNCs. **e,** Comparison of a GDP analogue bound ARF1 crystal structure with nascent ARF1. **f,** Comparison of crystal structures of GDP analog bound ARF1 and GDP bound ARF62. N-termini have been removed clarity. Molecular models with leucine residues of the C-terminal α-helix contributing to the myristoyl binding pocket are shown (top). Surface representation colored by lipophilicity of the molecular models on top are shown (bottom). **g,** AF3 prediction of ARF1 with the NACA-UBA domain as an alternative conformation explaining unidentified extra density. Surface representation of ARF1 colored by lipophilicity (-20, 0, 20 corresponding to turquoise, white and beige) in the bottom left highlighting the hydrophobic interaction site of the NACA-UBA domain. **h,** AF3 prediction of the NACA-UBA domain with ARF1 using amino acids 2-181 of ARF1 plus the two terminal V5 amino acids that are at the 60S exit as input. **i,** Distance calculation between the end of the V5 tag and the end of an 8 amino acid substrate bound to NMT1 at its alternative position. ARF1 and NAC are omitted for clarity. **j,** Comparison between the NAC globular domain positioning for the alternative NMT1 state and State 1 (purple and transparent). The overall rotation is indicated. **k,** Overlaid molecular models for NAC-METAP1 (PDB: 8P2K, right) and METAP2 (PDB: 8ONY, left) with the alternative NMT1 state. ARF1 and NAC are excluded for clarity. Source data are provided as a Source Data file.

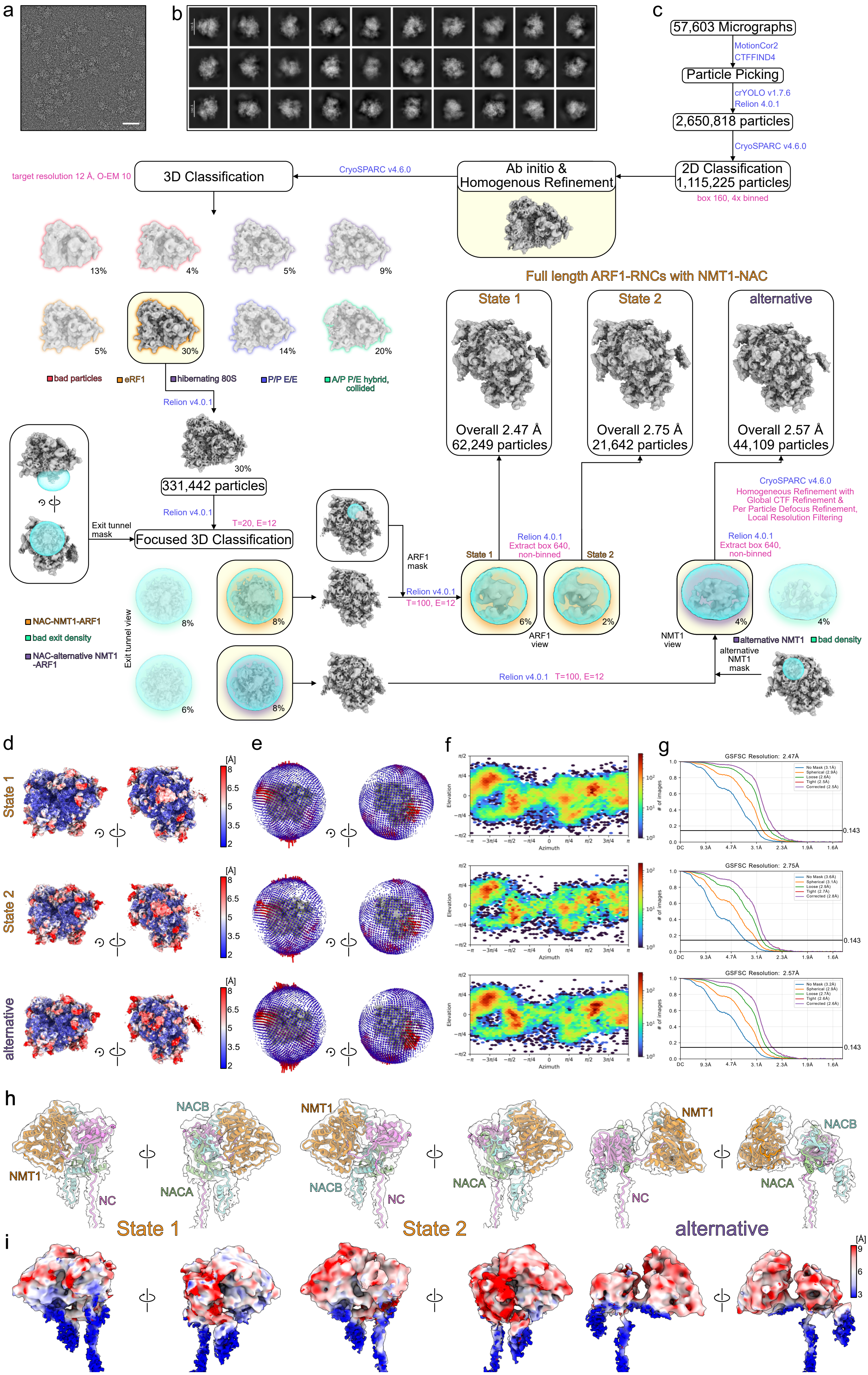

a
b
c
57,603 Micrographs
MotionCor2
CTFFIND4
Particle Picking
crYOLO v1.7.6
Relion 4.0.1
2,650,818 particles
CryoSPARC v4.6.0
2D Classification
1,115,225 particles
box 160, 4x binned
Ab initio &
Homogenous Refinement
CryoSPARC v4.6.0
3D Classification
target resolution 12 Å, O-EM 10
13%
4%
5%
9%
5%
30%
14%
20%
bad particles
eRF1
hibernating 80S
P/P E/E
A/P P/E hybrid, collided
Relion v4.0.1
30%
331,442 particles
Relion v4.0.1
T=20, E=12
Exit tunnel mask
Focused 3D Classification
NAC-NMT1-ARF1
bad exit density
NAC-alternative NMT1 -ARF1
Exit tunnel view
8%
8%
6%
8%
ARF1 mask
Relion v4.0.1
T=100, E=12
Full length ARF1-RNCs with NMT1-NAC
State 1
Overall 2.47 Å
62,249 particles
State 2
Overall 2.75 Å
21,642 particles
alternative
Overall 2.57 Å
44,109 particles
Relion 4.0.1
Extract box 640, non-binned
State 1
State 2
6%
2%
ARF1 view
CryoSPARC v4.6.0
Homogeneous Refinement with Global CTF Refinement & Per Particle Defocus Refinement, Local Resolution Filtering
Relion 4.0.1
Extract box 640, non-binned
4%
4%
NMT1 view
alternative NMT1
bad density
alternative NMT1 mask
Relion v4.0.1 T=100, E=12
d
e
f
g
State 1
State 2
alternative
[Å]
8
6.5
5
3.5
2
Elevation
Azimuth
# of images
GSFSC Resolution: 2.47Å
GSFSC Resolution: 2.75Å
GSFSC Resolution: 2.57Å
0.143
h
NMT1
NACB
NACA
NC
State 1
State 2
alternative
i
[Å]
9
6
3

Supplementary Fig. 8: Data processing of the full-length ARF1 RNC-NMT-NAC in vitro cryo-EM dataset. a, Representative, filtered micrograph with 35 nm scale bar. b, Representative 2D class averages for RNCs. **c,** cryo-EM data processing and sorting scheme. Selected classes from 3D classifications are framed. Relevant processing steps are highlighted, parameters are noted in pink and software used in blue. **d-g,** Validations and resolution determination for State 1, State 2 and the alternative ARF1-NMT1-NAC state. **d,** cryo-EM maps filtered and colored according to local resolution. **e,** 3D representations of the orientation distributions of final particles. **f,** 2D heatmap for particle viewing direction and numbers as generated by cryoSPARC. **g,** gold standard Fourier shell correlation curves (GSFSC, as generated by cryoSPARC) with 0.143 criterion for overall resolution estimation. **h,** Segmented, local resolution filtered densities and molecular models for the different states of NMT1-NAC-nascent full-length ARF1 exit-tunnel assemblies. **i,** Segmented densities shown in **h** colored according to local resolution.

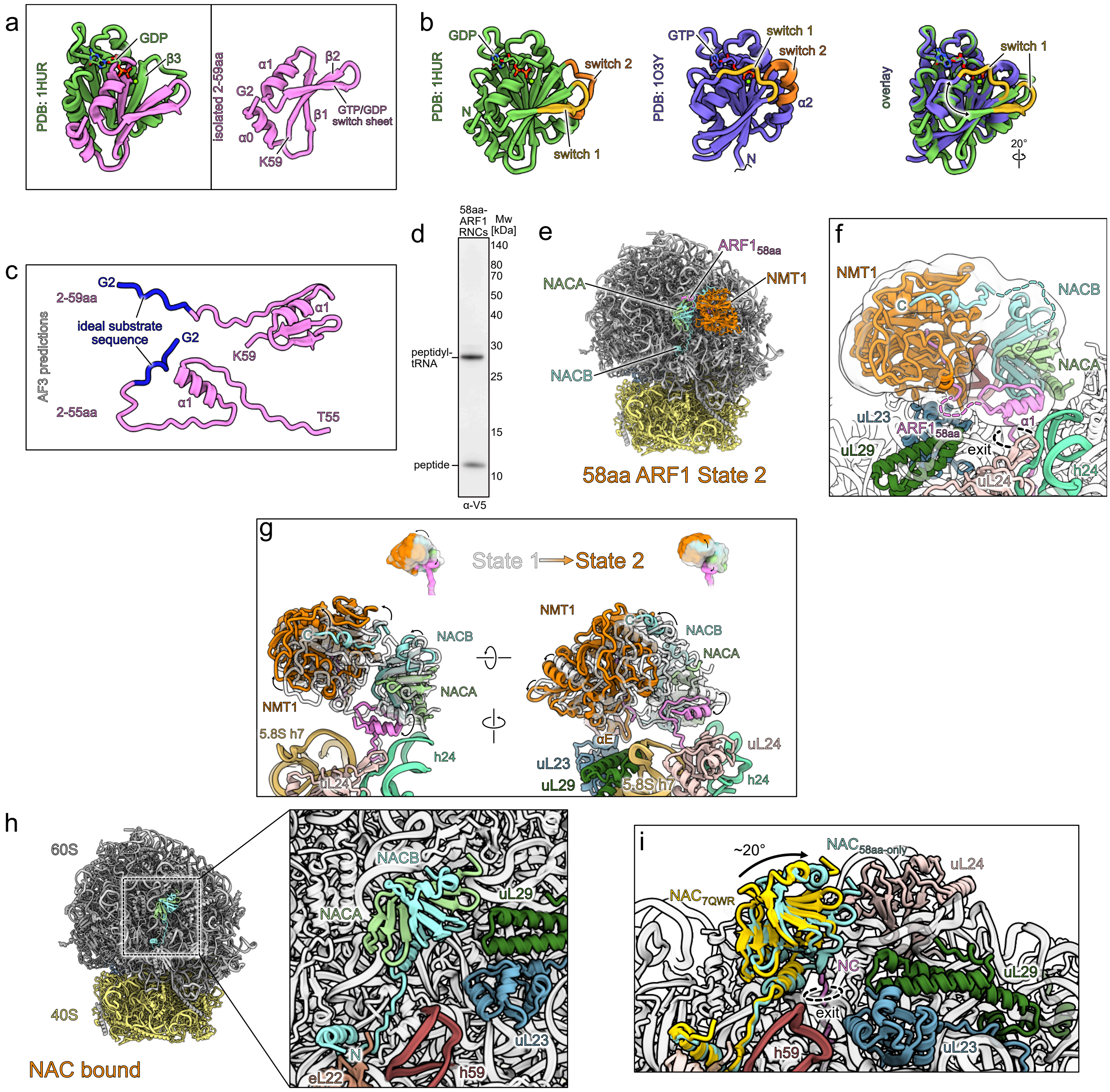

Supplementary Fig. 9: Details of the in vitro reconstituted NMT1-NAC 58 amino acid ARF1 intermediate RNC complexes. a, Molecular model of the GDP bound ARF1 (ref) in green with the first 58 amino acids (2-59aa) in pink (left) and the isolated first 58 amino acids with relevant secondary structure features annotated (right). **b,** Molecular models of GDP bound human ARF1[3] (left) as in a and GTP bound mouse ARF1[4] (middle) which share 100% amino acid identity as well as the overlay of both (right) with GTP/GDP switches 1 and 2 indicated in yellow and orange, respectively. GTP binding leads to complete formation of helix α2 and the GTP/GDP switch sheet shown in a becomes disorder. **c,** AF3 predictions of the ARF1 substrate used for the 58aa ARF1 RNCs. **d,** Western blot of purified 58aa-ARF1 RNCs. The nascent polypeptide was detected via the encoded V5 peptide. The shift of the peptide product (10.2 kDa) to ~27 kDa is indicative of intact peptidyl-tRNA and stable ribosome stalling by the hCMV peptide. The contrast was adjusted. **e,** Molecular model of NMT1-NAC bound 58aa-ARF1-State 2 RNCs. f, Close-up of the nascent 58aa-ARF1 nascent peptide engaged by NMT1 and NAC in the 58aa-ARF1 State 2. Transparent filtered, zoned density for the exit site assembly is shown. **g,** Comparison of 58aa-ARF1 State 1 and 2 with State 1 overlaid in gray and transparent. Arrows indicate the movement of individual factors and nascent 58aa-ARF1 from State 1 to 2. Cartoon representations in the right top corners summarize the overall movements. **h,** Overview of the entire ribosome (left) and the peptide exit site (right) for the NAC bound RNC subclass from the 58aa-ARF1 NMT1-NAC reconstitution dataset with relevant rRNA elements and ribosomal proteins highlighted. **i,** Comparison between NAC complex globular domain positioning for a signal sequence containing RNC (PDB: 7QWR) in yellow and the 58aa-ARF1 intermediate RNC in turquoise. Overlaid molecular models are shown and the rotation for the globular domain from the signal sequence RNC to the nascent 58aa-ARF1 RNC is indicated. Source data are provided as a Source Data file.

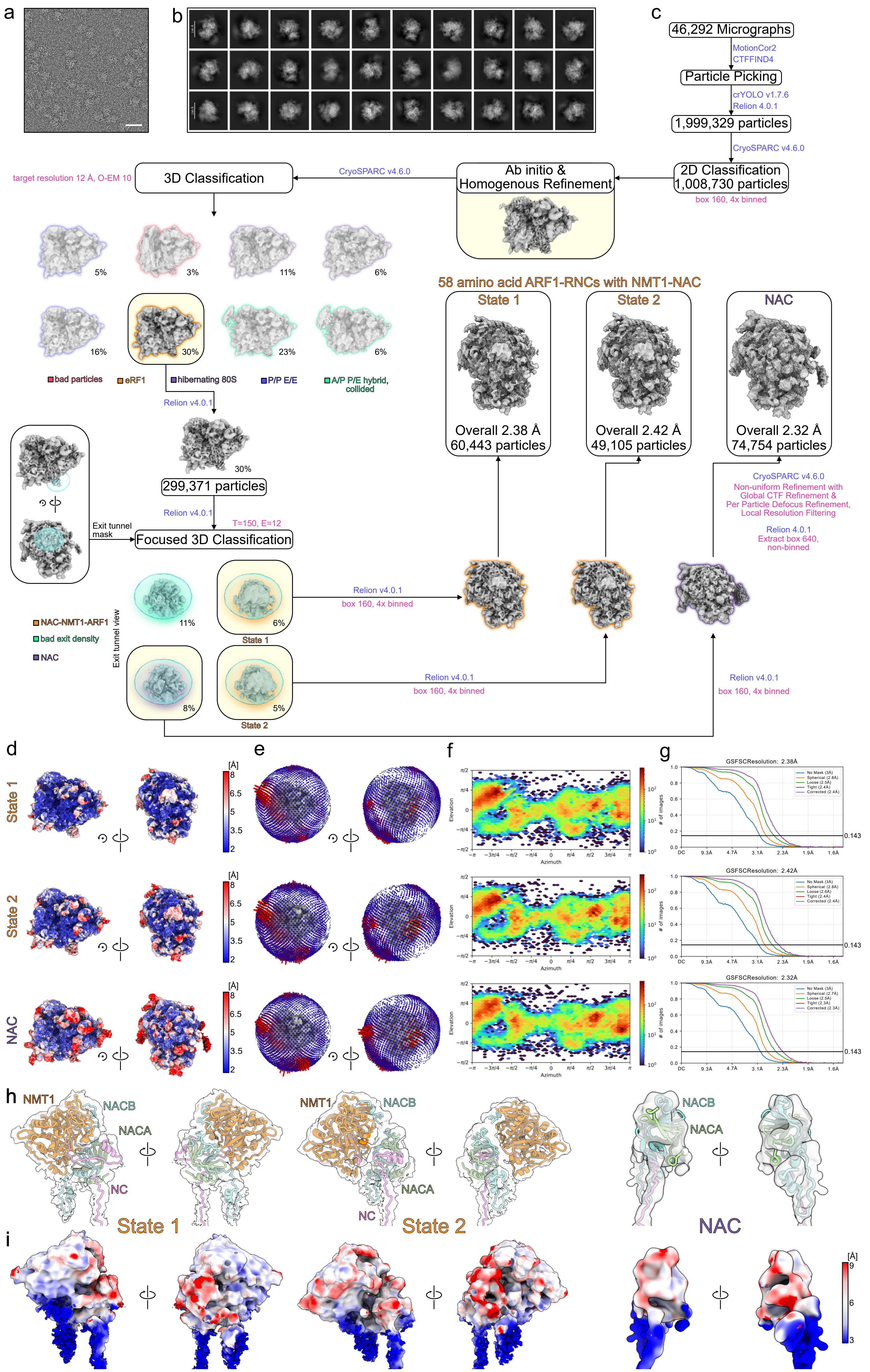

a
b
c
46,292 Micrographs
MotionCor2
CTFFIND4
Particle Picking
crYOLO v1.7.6
Relion 4.0.1
1,999,329 particles
CryoSPARC v4.6.0
2D Classification
1,008,730 particles
box 160, 4x binned
Ab initio &
Homogenous Refinement
CryoSPARC v4.6.0
3D Classification
target resolution 12 Å, O-EM 10
5%
3%
11%
6%
16%
30%
23%
6%
bad particles
eRF1
hibernating 80S
P/P E/E
A/P P/E hybrid, collided
Relion v4.0.1
30%
299,371 particles
Relion v4.0.1
T=150, E=12
Exit tunnel mask
Focused 3D Classification
NAC-NMT1-ARF1
bad exit density
NAC
Exit tunnel view
11%
6%
State 1
8%
5%
State 2
Relion v4.0.1
box 160, 4x binned
58 amino acid ARF1-RNCs with NMT1-NAC
State 1
Overall 2.38 Å
60,443 particles
State 2
Overall 2.42 Å
49,105 particles
NAC
Overall 2.32 Å
74,754 particles
CryoSPARC v4.6.0
Non-uniform Refinement with Global CTF Refinement & Per Particle Defocus Refinement, Local Resolution Filtering
Relion 4.0.1
Extract box 640, non-binned
d
e
f
g
State 1
State 2
NAC
[Å]
8
6.5
5
3.5
2
Elevation
Azimuth
# of images
GSFSCResolution: 2.38Å
GSFSCResolution: 2.42Å
GSFSCResolution: 2.32Å
No Mask
Spherical
Loose
Tight
Corrected
0.143
h
NMT1
NACB
NACA
NC
State 1
State 2
NAC
i
[Å]
9
6
3

Supplementary Fig. 10: Data processing of the 58 amino acid ARF1 intermediate RNC-NMT-NAC in vitro cryo-EM dataset. a, Representative, filtered micrograph with 35 nm scale bar. **b,** Representative 2D class averages for RNCs. **c,** cryo-EM data processing and sorting scheme. Selected classes from 3D classifications are framed. Relevant processing steps are highlighted, parameters are noted in pink and software used in blue. **d-g,** Validations and resolution determination for NTM1-NAC State 1, State 2 and the NAC only state. **d,** cryo-EM maps filtered and colored according to local resolution. e, 3D representations of the orientation distributions of final particles. **f,** 2D heatmap for particle viewing direction and numbers as generated by cryoSPARC. **g,** gold standard Fourier shell correlation curves (GSFSC, as generated by cryoSPARC) with 0.143 criterion for overall resolution estimation. **h,** Segmented, local resolution filtered densities and molecular models for the two NMT1-NAC and the NAC only-nascent intermediate ARF1 exit-tunnel assemblies. **i,** Segmented densities shown in **h** colored according to local resolution.

Supplementary References

1 Hein, M. Y. *et al.* A human interactome in three quantitative dimensions organized by stoichiometries and abundances. *Cell* **163**, 712-723 (2015). https://doi.org/10.1016/j.cell.2015.09.053

2 Menetrey, J., Macia, E., Pasqualato, S., Franco, M. & Cherfils, J. Structure of Arf6-GDP suggests a basis for guanine nucleotide exchange factors specificity. *Nat Struct Biol* **7**, 466-469 (2000). https://doi.org/10.1038/75863

3 Amor, J. C., Harrison, D. H., Kahn, R. A. & Ringe, D. Structure of the human ADP-ribosylation factor 1 complexed with GDP. *Nature* **372**, 704-708 (1994). https://doi.org/10.1038/372704a0

4 Shiba, T. *et al.* Molecular mechanism of membrane recruitment of GGA by ARF in lysosomal protein transport. *Nat Struct Biol* **10**, 386-393 (2003). https://doi.org/10.1038/nsb920
