# Supplementary material for: Structural basis of co-translational N-myristoylation in humans
Source: Nat Commun. 2026 Jan 23;17:1191. doi: 10.1038/s41467-025-67962-4 (PMC12858966; doi:10.1038/s41467-025-67962-4)
Supplement: Supplementary file 3 — Reporting Summary [file 41467_2025_67962_MOESM3_ESM.pdf]

nature portfolio

Corresponding author(s): Roland Beckmann, Thierry Meinnel

Last updated by author(s): Nov 27, 2025

Reporting Summary

Nature Portfolio wishes to improve the reproducibility of the work that we publish. This form provides structure for consistency and transparency in reporting. For further information on Nature Portfolio policies, see our Editorial Policies and the Editorial Policy Checklist.

Statistics

For all statistical analyses, confirm that the following items are present in the figure legend, table legend, main text, or Methods section.

n/a	Confirmed	
☐	☒	The exact sample size (*n*) for each experimental group/condition, given as a discrete number and unit of measurement
☐	☒	A statement on whether measurements were taken from distinct samples or whether the same sample was measured repeatedly
☐	☒	The statistical test(s) used AND whether they are one- or two-sided<br>*Only common tests should be described solely by name; describe more complex techniques in the Methods section.*
☒	☐	A description of all covariates tested
☒	☐	A description of any assumptions or corrections, such as tests of normality and adjustment for multiple comparisons
☐	☒	A full description of the statistical parameters including central tendency (e.g. means) or other basic estimates (e.g. regression coefficient) AND variation (e.g. standard deviation) or associated estimates of uncertainty (e.g. confidence intervals)
☒	☐	For null hypothesis testing, the test statistic (e.g. *F*, *t*, *r*) with confidence intervals, effect sizes, degrees of freedom and *P* value noted<br>*Give P values as exact values whenever suitable.*
☒	☐	For Bayesian analysis, information on the choice of priors and Markov chain Monte Carlo settings
☒	☐	For hierarchical and complex designs, identification of the appropriate level for tests and full reporting of outcomes
☒	☐	Estimates of effect sizes (e.g. Cohen's *d*, Pearson's *r*), indicating how they were calculated

Our web collection on statistics for biologists contains articles on many of the points above.

Software and code

Policy information about availability of computer code

Data collection	EPU v3.3.1 (Thermo Fisher Scientific) software suite was used for cryo-EM data collection.
Data analysis	Cryo-EM data was processed using MotionCor2 v1.4.0 and CTFFIND4 v4.1.13 for motion correction and initial CTF parameter determination, respectively. For particle picking crYOLO v1.7.6 was used. 2D and 3D classifications, 3D refinements as well as general cryo-EM data processing was done using the Relion v4.0.1 and cryoSPARC v4.4.0 or v4.6.0 software suites. Molecular models were built using Coot v0.9.8. Phenix v1.21.2-5419 was used for real-space refinement of molecular models. Models and density maps were analyzed and visualized using ChimerX v1.8. Statistical analysis of gel band intensities was performed using GraphPad Prism v10.2.3.

For manuscripts utilizing custom algorithms or software that are central to the research but not yet described in published literature, software must be made available to editors and reviewers. We strongly encourage code deposition in a community repository (e.g. GitHub). See the Nature Portfolio guidelines for submitting code & software for further information.

Data

Policy information about availability of data

All manuscripts must include a data availability statement. This statement should provide the following information, where applicable:

- Accession codes, unique identifiers, or web links for publicly available datasets
- A description of any restrictions on data availability
- For clinical datasets or third party data, please ensure that the statement adheres to our policy

Cryo-EM maps and molecular models generated in the study were deposited at the Electron Microscopy Data Bank (EMDB) or the Protein Data Bank (PDB), respectively, and are accessible via the following codes: EMD-52581 [https://www.ebi.ac.uk/emdb/EMD-52581] and 9I2D [https://doi.org/10.2210/pdb9i2d/pdb] (NMT1-NAC bound human RNC with 10 amino acid ARF1-linker); EMD-52582 [https://www.ebi.ac.uk/emdb/EMD-52582] and 9I2E [https://doi.org/10.2210/pdb9i2e/pdb] (NMT1-NAC bound human ribosome (combined translational states)); EMD-53230 [https://www.ebi.ac.uk/emdb/EMD-53230] and 9QLO [https://doi.org/10.2210/pdb9qlo/pdb] (NMT1-NAC bound human RNC with full length ARF1 - State 1); EMD-53231 [https://www.ebi.ac.uk/emdb/EMD-53231] and 9QLP [https://doi.org/10.2210/pdb9qlp/pdb] (NMT1-NAC bound human RNC with full length ARF1 - State 2); EMD-53232 [https://www.ebi.ac.uk/emdb/EMD-53232] and 9QLQ [https://doi.org/10.2210/pdb9qlq/pdb] (NMT1-NAC bound human RNC with full length ARF1 – alternative State); EMD-54528 [https://www.ebi.ac.uk/emdb/EMD-54528] and 9S3B [https://doi.org/10.2210/pdb9s3b/pdb] (NMT1-NAC bound human RNC with 58 amino acid ARF1-linker - State 1); EMD-54529 [https://www.ebi.ac.uk/emdb/EMD-54529] and 9S3C [https://doi.org/10.2210/pdb9s3c/pdb] (NMT1-NAC bound human RNC with 58 amino acid ARF1-linker - State 2); EMD-54530 [https://www.ebi.ac.uk/emdb/EMD-54530] and 9S3D [https://doi.org/10.2210/pdb9s3d/pdb] (NAC bound human RNC with 58 amino acid ARF1-linker). Source data are provided with this paper.

Research involving human participants, their data, or biological material

Policy information about studies with human participants or human data. See also policy information about sex, gender (identity/presentation), and sexual orientation and race, ethnicity and racism.

Reporting on sex and gender	n/a
Reporting on race, ethnicity, or other socially relevant groupings	n/a
Population characteristics	n/a
Recruitment	n/a
Ethics oversight	n/a

Note that full information on the approval of the study protocol must also be provided in the manuscript.

Field-specific reporting

Please select the one below that is the best fit for your research. If you are not sure, read the appropriate sections before making your selection.

☒ Life sciences ☐ Behavioural & social sciences ☐ Ecological, evolutionary & environmental sciences

For a reference copy of the document with all sections, see nature.com/documents/nr-reporting-summary-flat.pdf

Life sciences study design

All studies must disclose on these points even when the disclosure is negative.

Sample size	Statistical determination of sample sizes for in vivo and in vitro experiments was not performed and is not applicable in this study. For cryo-EM data collections the number of micrographs varied (13,000-57,000) depending on the sample. Here, collection size was chosen to allow for extensive 2D and 3D classification for each sample.
Data exclusions	No data was intentionally excluded, except for cryo-EM 2D classes that did not show distinct features of 80S ribosomes.
Replication	In vitro and in vivo data could be reproduced successfully by repetition. Cryo-EM data for each sample was only collected once.
Randomization	For resolution determination of cryo-EM reconstructions the "Gold Standard" Fourier shell correlation (FSC) was used. Particles are randomly divided into halves and two independent reconstructions are performed which are used for FSC calculation. For anything else in this study randomization does not apply.
Blinding	Group allocation was not performed. Blinding does not apply and is not relevant for this study.<br>Cryo-EM data processing, including particle division for FSC calculation, are computer-based calculations and processes, and unbiased in nature.

Reporting for specific materials, systems and methods

We require information from authors about some types of materials, experimental systems and methods used in many studies. Here, indicate whether each material, system or method listed is relevant to your study. If you are not sure if a list item applies to your research, read the appropriate section before selecting a response.

Materials & experimental systems

n/a	Involved in the study
☐	☒ Antibodies
☐	☒ Eukaryotic cell lines
☒	☐ Palaeontology and archaeology
☒	☐ Animals and other organisms
☒	☐ Clinical data
☒	☐ Dual use research of concern
☒	☐ Plants

Methods

n/a	Involved in the study
☒	☐ ChIP-seq
☒	☐ Flow cytometry
☒	☐ MRI-based neuroimaging

Antibodies

Antibodies used	mouse anti-V5 - abcam #ab27671, clone SV5-Pk1, lot 1025365-10, dilution 1:2,000<br>mouse anti-FLAG M2 HRP-conjugated - Sigma Aldrich #A8592,clone M2, lot 0000259468, dilution 1:10,000<br>rabbit anti-GAPDH - ProteinTech #10494-1-AP, lot 00055216, diluted 1:10,000<br>goat anti-mouse IgG HRP-conjugated - dianova #115-035-003, lot 158670, dilution 1:10,000<br>goat anti-rabbit IgG HRP-conjugated - Sigma Aldrich #A0545, lot 069M4835V, dilution 1:10,000<br>rabbit anti-NACA - Wiedmann, B. et al. Nature 1994, dilution 1:2,000<br>rabbit anit-RPS6 (eS6) - Cell Signaling Technology #2217, lot 13, dilution 1:1,000<br>rabbit anit-RPS10 (eS10) - abcam #ab151550, clone EPR8545, lot GR3396422-8, dilution 1:1,000<br>goat anti-rabbit IgG HRP-conjugated- Sigma Aldrich #A6154, lot SLBG7201V, dilution 1:10,000<br>monoclonal recombinant human anti-DYKDDDDK HRP-conjugated - Miltenyi Biotec, #130-101-572, clone REA216, lot 5160209288
Validation	mouse anti-V5 - Validated by the manufacturer for WB: https://www.abcam.com/en-us/products/primary-antibodies/v5-tag-antibody-sv5-pk1-ab27671<br>mouse anti-FLAG M2 HRP-conjugated - Validated by the manufacturer for WB: https://www.sigmaaldrich.com/DE/en/product/sigma/a8592<br>rabbit anti-GAPDH - ProteinTech - Validated by the manufacturer for WB: https://www.ptglab.com/products/GAPDH-Antibody-10494-1-AP.htm<br>goat anti-mouse IgG HRP-conjugated - Validated by the manufacturer for WB: https://www.dianova.com/en/shop/115-035-003-goat-igg-anti-mouse-igg-hl-hrpo-minx-none/<br>goat anti-rabbit IgG HRP-conjugated - Sigma Aldrich #A0545 - Validated by the manufacturer for WB: https://www.sigmaaldrich.com/DE/en/product/sigma/a0545?msockid=25ae4c81361d63810d3f5fbf377662cf<br>rabbit anti-NACA - Wiedmann, B. et al. Nature 1994: Figure 2c and Methods<br>rabbit anit-RPS6 (eS6) - Validated by the manufacturer for WB: https://www.cellsignal.com/products/primary-antibodies/s6-ribosomal-protein-5g10-rabbit-monoclonal-antibody/2217<br>rabbit anit-RPS10 (eS10) - Validated by the manufacturer for WB: https://www.abcam.com/en-us/products/primary-antibodies/rps10-antibody-epr8545-ab151550<br>goat anti-rabbit IgG HRP-conjugated- Sigma Aldrich #A6154 - Validated by the manufacturer for WB: https://www.sigmaaldrich.com/DE/en/product/sigma/a6154?msockid=25ae4c81361d63810d3f5fbf377662cf<br>monoclonal recombinant human anti-DYKDDDDK HRP-conjugated - Validated by the manufacturer for WB: https://www.miltenyibiotec.com/UN-en/products/dykddddk-antibody-reafinity-rea216.html#conjugate=vio-b515:size=100-tests-in-200-ul

Eukaryotic cell lines

Policy information about cell lines and Sex and Gender in Research

Cell line source(s)	HEK293T - ATCC #CRL-3216<br>HeLa S3 - Sigma Aldrich #87110901<br>Expi293F - Thermo Fisher Scientific #A14527<br>HEK293 - ATCC #CRL-1573
Authentication	None of the cell lines were authenticated.
Mycoplasma contamination	Cell lines were regularly tested for mycoplasma by PCR. No contamination was detected.
Commonly misidentified lines<br>(See ICLAC register)	No commonly misidentified cell lines were used in this study.

Plants

Seed stocks	n/a
Novel plant genotypes	n/a
Authentication	n/a
